# High enthalpy storage thermoset network with giant stress and energy output in rubbery state

**DOI:** 10.1038/s41467-018-03094-2

**Published:** 2018-02-13

**Authors:** Jizhou Fan, Guoqiang Li

**Affiliations:** Department of Mechanical & Industrial Engineering, Louisiana State University, Baton Rouge, LA 70803 USA

## Abstract

Low output in stress and energy in rubbery state has been a bottleneck for wide-spread applications of thermoset shape memory polymers (SMPs). Traditionally, stress or energy storage in thermoset network is through entropy reduction by mechanical deformation or programming. We here report another mechanism for energy storage, which stores energy primarily through enthalpy increase by stretched bonds during programming. As compared to entropy-driven counterparts, which usually have a stable recovery stress from tenths to several MPa and energy output of several tenths MJ/m^3^, our rubbery network achieved a recovery stress of 17.0 MPa and energy output of 2.12 MJ/m^3^ in bulk form. The giant stress and energy release in the rubbery state will enhance applications of thermoset SMPs in engineering structures and devices.

## Abstract (web summary)

Energy storage in thermoset shape memory polymers happens through entropy reduction during the programming step, but low energy release is known to be a bottleneck for wide-spread application. Here, the authors show a thermoset network that stores energy primarily through enthalpy increase by bond length change, which leads to an improved energy output.

## Introduction

Shape memory polymers (SMPs) have been a topic of intensive research for years^1–10^. In addition to shape memory, which means a deformed temporary shape can return to its original permanent shape upon stimulation, such as heat^11^, light^12^, moisture^13^, pH^14^, etc., SMPs can also release stress if free shape recovery is not allowed^1^. The fact that SMPs can memorize both shape and stress has rendered them with many potential applications such as actuators, self-healing, sealants, morphing structures, stent, suture, soft robot, smart textile, etc.^1–10^. While many stimuli approaches have been used in SMPs such as host–guest transition^15^, anisotropic–isotropic transition^7^, etc., thermal transition has been the most popular method because some other methods such as electricity and magnetic field also cause indirect heating^2,16^. Heat-induced shape memory effect is triggered primarily by glass/vitrification transition and melt/crystallization transition^7^. For thermally triggered SMPs, a bottleneck is the low recovery stress^17^. In the several thermoset SMP systems reported as having very high stabilized recovery stress in the literatures, the majority exhibit stabilized recovery stress from tenths MPa to several MPa^1–10^. However, in many applications, higher recovery stress is needed, or higher recovery stress leads to better results such as higher healing efficiency in self-healing applications^18^.

For classical SMPs with glass transitions, entropy has been identified as the driving force for shape or stress recovery^1,2^. During the transition from glassy state to rubbery state for amorphous thermoset polymers, it is not uncommon to see one to two orders decrease in the modulus of the polymers. The dramatic reduction in modulus through the transition is necessary for the SMP to demonstrate excellent shape recovery; however, it sacrifices stress recovery. The flexible rubbery state suggests that the SMP can only release a low stress. In other words, for higher recovery stress, the SMP in rubbery state must be stiffer; however, it may suffer from lower shape memory. Therefore, for entropy-driven SMPs with thermal transitions, the contradictory requirement between recovery strain and recovery stress renders most thermoset SMPs with excellent shape memory but poor stress memory. Therefore, it is a grand challenge on how to increase the stress memory while maintaining excellent shape memory. Ideas other than entropy driven must be sought.

The enhancement of stress memory can be achieved by enriching energy storage during programming. Based on the basic thermodynamics, Δ*G* = Δ*H* − *T*Δ*S*, where Δ*G*, Δ*H*, and Δ*S* are the change of Gibbs free energy, enthalpy, and entropy, respectively, and *T* is the absolute temperature; hence, the stored energy consists of both entropy and enthalpy. Obviously, stress recovery and energy output depend on the energy input during programming and the energy storage in the temporary shape after programming^17^. Because entropy elasticity is the acknowledged driving force for shape and stress memory in previous SMPs, we believe that storing enthalpy during programming should be a way to further increase the recovery stress and energy output.

Here, we synthesized and characterized a thermoset SMP made of epoxy (EPON 826) cured by a rigid isophorone diamine (IPD), which stores energy primarily through enthalpy increase by bond length change. As compared to entropy-driven thermoset SMPs, which usually have a stable recovery stress from tenths to several MPa and energy output of several tenths MJ/m^3^, our enthalpy storage thermoset SMP achieved a stable recovery stress of 17.0 MPa and energy output of 2.12 MJ/m^3^ in rubbery state and in bulk form. This study may open up opportunities for applications of thermoset SMPs in engineering structures and devices which need large recovery stress and/or high energy output.

## Results

### Stress and energy storage and recovery

To obtain a thermoset network with high recovery stress and energy output through enthalpy storage, a commercially available epoxy (EPON 826) was reacted with a rigid diamine named IPD, which can provide a large steric hindrance. Detailed synthesis procedure for the EPON–IPD network is described in Methods. The large steric hindrance can ensure enthalpy increase during programming and also can reduce the stress relaxation in rubbery state (Supplementary Fig. 12), which enhances energy output during partially constrained shape recovery test.

**Fig. 1:**
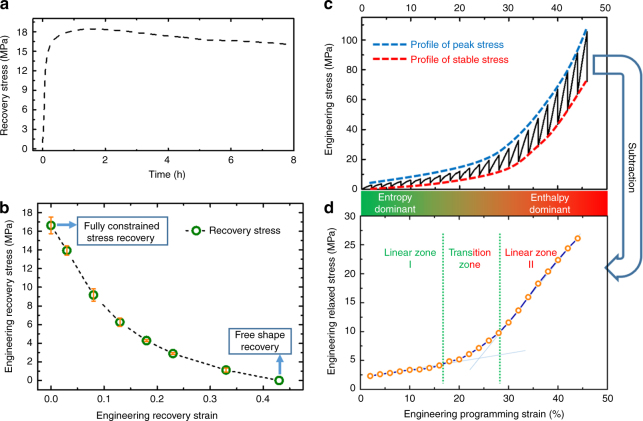
The stress and energy storage and recovery behavior. **a** The fully constrained stress recovery profile in rubbery state (recovered at 170 °C for 8 h; the glass transition zone is between 140 and 160 °C; see Supplementary Fig. 3) for a sample compression programmed with 45% pre-strain at a strain rate of 0.5 mm/mm/min and temperature of 170 °C. Detailed compression programming and fully constrained shape recovery test can be found in Supplementary Methods. The recovery stress in the rubbery state is about 17.87 MPa at 1.0 h, 17.0 MPa at 1.5 h, and 16.07 MPa at 8 h. **b** The relationship between the recovery stress and recovery strain (the recovery stress was taken at 1.5 h). The test procedure is given in Supplementary Methods. The free shape recovery ratio was 99.9%. The energy output, which is calculated based on the area of the recovery stress–strain curve, is about 2.12 MJ/m^3^. **c** The stepwise iso-strain programming profile. In order to elucidate the different modes for energy storage, step-wise iso-strain compression programming was conducted. In each step of loading, the strain increases; the stress then relaxes while holding the strain constant, which completes the one loading-relaxation cycle. In each step, the sample was compressed to 2% strain and then let it relax for 4 min. The detailed test procedure is shown in Supplementary Fig. 16 and the strain rate effect is illustrated in Supplementary Fig. 13. **d** The change of programming stress after relaxation, or stored stress, with programming strain. The stored stress increases as the programming strain increases, which suggests that more energy input leads to more energy storage, and thus higher recovery strain and higher recovery stress. The stored energy is calculated by the area of this relaxation stress–strain curve, which is 4.10 MJ/m^3^

Figure 1a shows the fully constrained stress recovery test results. The maximum recovery stress, as high as 17 MPa in rubbery state, was obtained and largely maintained. The recovery stress versus recovery strain through partially constrained shape recovery test is plotted in Fig. 1b. Based on Fig. 1b, more than 6 MPa stress can still be maintained even when the programmed sample with 45% pre-strain is allowed to recover 10% of strain. This stress is adequate to drive crack closure in real world applications^18^. Based on this recovery stress–recovery strain curve, the energy output, i.e., the area included by the recovery stress–recovery strain curve, is calculated to be 2.12 MJ/m^3^, which is much higher than other thermoset SMPs or even elastically deformed metals, and is even comparable to some shape memory alloys (SMAs), as given in Supplementary Table 2.

Figure 1c shows a stepwise iso-strain programming experiment or stepwise stress relaxation test in order to reveal the energy storage mechanism in this thermoset network. We conducted this experiment because stress relaxation is a mechanism for energy storage during programming^19^. In each step, the sample was compressed to 2% strain and then let it relax for 4 min. Subtracting the stabilized stress (stress after relaxation) from the peak stress in each step, the stress relaxation profile is obtained, as shown in Fig. 1d. Two distinct linear zones, separated by a transition zone (TZ), can be identified. The slope of the second linear zone, which represents the relaxed modulus of the polymer, is much higher than that of the first zone. This is a physical evidence that this thermoset network has a giant recovery stress. The three zones in Fig. 1d indicate that the energy storage follows two different mechanisms during the programming process. Proved by Supplementary Discussion, in Linear Zone I (LZ1), the energy is stored through entropy reduction. In the TZ, the energy storage is through both entropy reduction and enthalpy increase, but gradually with more and more share by enthalpy as the programming strain increases. In Linear Zone II (LZ2), the energy is primarily stored by increase in enthalpy. From Fig. 1d, the stored energy, which is the area included by the relaxation stress–strain curve, is calculated to be 4.10 MJ/m^3^. Therefore, the energy output efficiency is 2.12 MJ/m^3^/4.10 MJ/m^3^ = 51.71%.

The energy storage mechanism can also be understood at the molecular level. The synthesized EPON–IPD network can be treated as a continuous elastic body in rubbery state when the unreacted residual monomers and defects are neglected. From low to high energy state, only three molecular structural parameters, which are the dihedral angle, bond length, and bond angle, can be changed during the programming process^20^. The dihedral angle can be changed by bond rotation; while the change in bond length and bond angle might happen by stretching, compressing, or bending the chemical bonds. In general, bond angle is determined by the type of orbiters such as *sp*2, *sp*3, etc., and it is the most difficult parameter to change. Therefore, it is assumed that bond angles are constant in this study. During mechanical deformation (programming), the parameter with low energy state can be changed first, which is the dihedral angle. Each change in the dihedral angle leads to an ordered or aligned conformational configuration of the network along the loading direction, or entropy decrease, which corresponds to the LZ1 in Fig. 1d. With further deformation, the dihedral angle change becomes more difficult because the free volume is reduced, and the available conformational configurations become less. Therefore, the deformation is shifted gradually towards bond length change. Clearly, bond length changes do not render conformational entropy changes, but they increase enthalpy. This gradual shift from entropy decrease to enthalpy increase corresponds to the TZ in Fig. 1d. With higher programming strain, the energy will be primarily stored by the bond length change, i.e., enthalpy increases, leading to the LZ2 in Fig. 1d. The bond length starts to change in TZ and change more in LZ2 are confirmed by the Raman spectroscopy and near edge X-ray absorption fine structure spectroscopy (NEXAFS) as shown in Supplementary Figs. 17 and 18, respectively.

### Characterization of enthalpy release

**Fig. 2:**
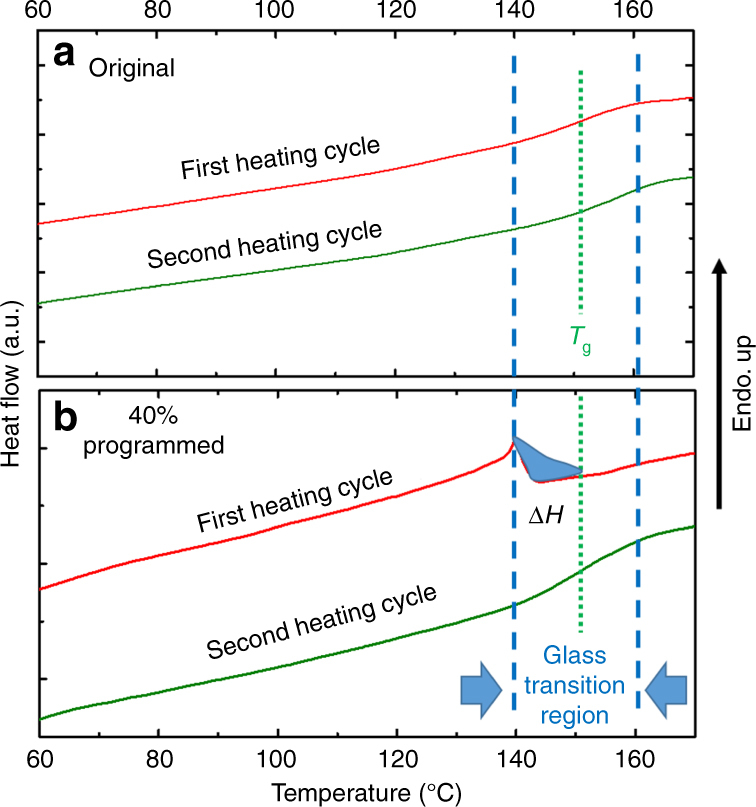
The enthalpy release during the free shape recovery process by DSC. **a** The DSC test results for the original SMP after synthesis. To avoid the post-curing effect and to match the thermal history with the programmed sample, the as prepared original SMP sample was heated at 170 °C for over 1 h before the DSC test. The typical glass transition curve, glass transition region, and glass transition temperature can be identified in the second heating cycle. **b** The DSC test results for the 40% compressive strain programmed sample. An inverse peak emerges during the first heating cycle in the pre-*T*_g_ region. The enthalpy release is confirmed by this first order transition. This is due to the retreat of stretched bonds formed during programming, which leads to release of the stored enthalpy, and the enthalpy release is Δ*H* = −2.85 J/g. The negative sign means the release of enthalpy. After release of the stored enthalpy, the second heating cycle also presents the classical second order glass transition curve. Based on the density of the sample, the specific enthalpy storage is 3.25 MJ/m^3^. Determination of the end points and baseline for calculating the enthalpy can be found in Supplementary Fig. 4

Figure 2 confirms enthalpy release during free shape recovery by differential scanning calorimetry (DSC) tests. Two thermal cycles were conducted for the un-deformed (control) and 40% compression strain programmed samples. Both samples show the same glass transition region in the second heating curve, because the first heating cycle has eliminated the history of programming. For the programmed sample, a high enthalpy release is confirmed by the inverse peak presenting in the first heating curve. The release starts at the on-set point of the glass TZ sharply. Considering both the baseline shift and the normal glass transition (Supplementary Fig. 4), the total specific enthalpy released by the stretching bond is −2.85 J/g. The negative sign means energy release. Considering that the density of the sample is 1.142 g/cm^3^, the enthalpy release density is 3.25 MJ/m^3^. Compared with the total energy stored in the 40% pre-strain programmed sample, which is 3.59 MJ/m^3^ calculated by integration from 0 to 40% strain in Fig. 1d, it is found that 90.5% (3.25 MJ/m^3^/3.59 MJ/m^3^) of the energy stored is in the form of enthalpy.

### Mechanism for enthalpy storage

**Fig. 3:**
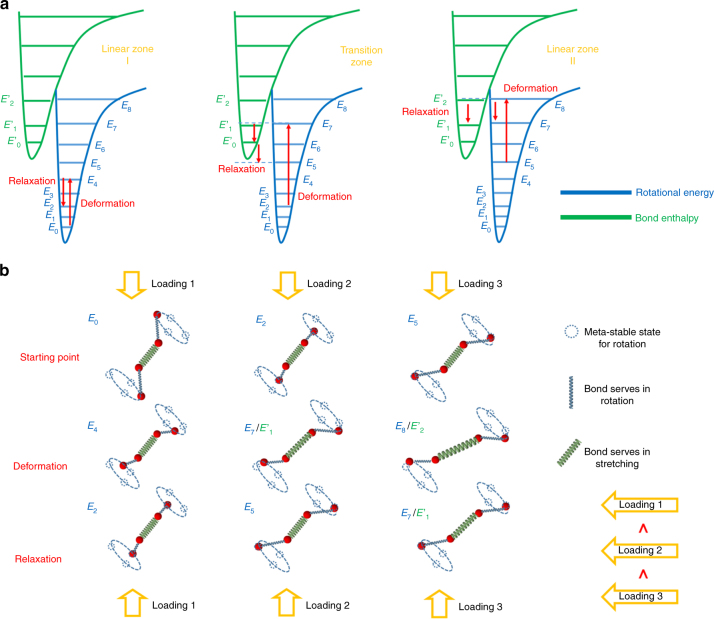
The energetical, structural, and conformational characteristics. During compression programming, the energetical, structural, and conformational evolution can be characterized by: **a** the energetical evolution corresponding to linear zone I (LZ1), transition zone (TZ), and linear zone II (LZ2). Deformation excites the energy to a higher level, most likely an unstable energy state; and after structural or stress relaxation, retreats to a local lower energy level, leading to meta-stable state. For example, in the LZ2, deformation excites the rotation energy level from *E*_5_ to *E*_8_, and relaxation retreats the energy level in terms of bond enthalpy to *E*_1_′. **b** The structural and conformational evolution corresponding to LZ1, TZ, and LZ2. The blue springs stand for rotating bonds and the green springs represent stretching bonds. The dashed circles are the possible locally meta-stable positions for the rotating bonds. Under loading 1, only bond rotation happens during both deformation and relaxation. Under loading 2, which is larger than loading 1, both bond rotation and stretching can happen during the deformation. However, the stretched bonds retreat during the relaxation. Under loading 3, which is the highest loading, the stretched bond can be stabilized in a certain conformation. The simplification made here is that the rotating bonds (blue springs) are fixed length during the deformation and the relaxation. The reality is that the rotating bonds can also be stretched

Figure 3 illustrates the relationship between deformation (energy input) and relaxation (energy storage) in different zones. Counter-intuitively, the compressive deformation does not shorten the bond length; instead, the bonds are stretched as shown in the schematic in Fig. 3b. In LZ1 in Fig. 1d, the deformation and relaxation are only related to the bond rotation as shown in Fig. 3a. With the increase in deformation, the total energy is excited to an energy level between the bond rotation energy and bond stretch energy. Because structural relaxation accompanies deformation, the total energy, after structural relaxation, assumes its stable energy state similar to the rotational energy state, and thus the bond length returns to its original length. With further increase in deformation, the total energy will gradually assume a higher energy state, away from the rotation energy state, but towards the bond stretch energy state, which leads to the TZ in Fig. 1d. With even further increase in compression deformation, the stabilized total energy is more towards the bond stretch energy, which is LZ2 in Fig. 1d. As a result of the enthalpy increase, around 43.8 MPa of internal stress can be stored by the stretched bonds; see calculation in Supplementary Discussions.

During the compressive deformation, the polymer network is in a non-equilibrium state at any instant. The stress relaxation is coupled with deformation. At each increment of deformation, the total free energy is excited to a higher level, most likely unstable. Due to the coupling of structural or stress relaxation, the excited energy level is relaxed back to a local energy well, to minimize the total free energy.

### Conformational or/and structural ball model

**Fig. 4:**
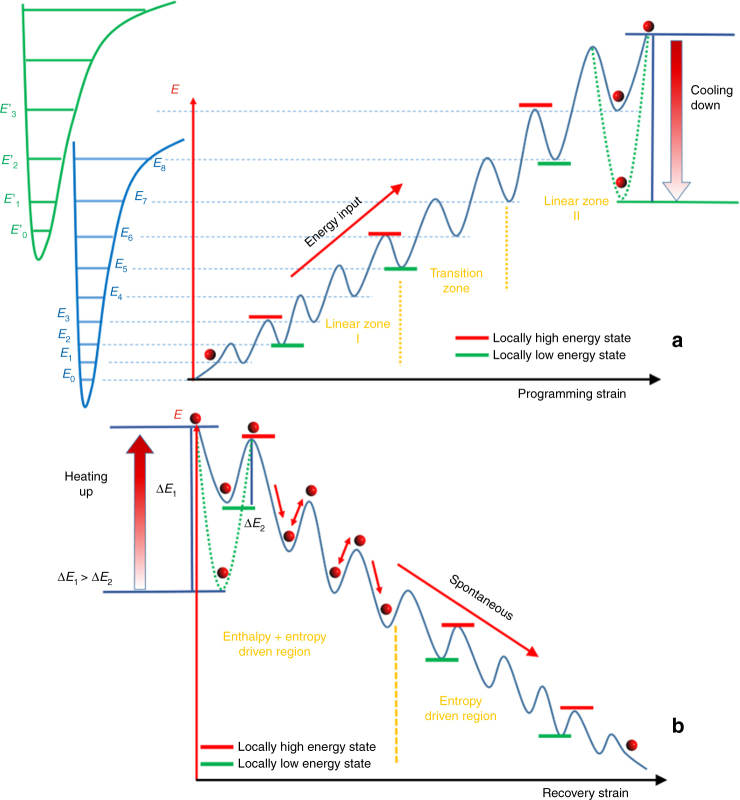
The multiple energy well model for amorphous thermoset shape memory polymers. **a** Programming. During programming at temperature above the glass transition zone, the network climbs up an energy hill with local energy well (or dip) (blue line) for local, meta-stale states. At the end of programming (after cooling and unloading), a deep energy well (dashed green line) is formed and thus the network is in a locked, non-equilibrium state. **b** Recovery. Energy input, such as heating, is needed to drive the cold energy well (dashed green line) back to the hot energy well (solid blue line) and help the CSBs (red circles) jump out of the final energy well, roll down the energy hill, and achieve shape recovery without external constraint, or stress recovery with external constraint

Figure 4a visualizes these natural characteristics hidden in the programming process. Each instantaneous non-equilibrium state is regarded as a locally high energy state and each instantaneous equilibrium state is regarded as a locally low energy state, the so called meta-stable state. This can be demonstrated by an analogy of a ball resting on an energy hill with many energy wells or dips. The physical meaning for the movement of the ball can be understood as a change of the conformation or structure. Hence, the ball is named as a conformational or/and structural ball (CSB). Each apex of the well corresponds to a local high energy state (non-equilibrium); each valley of the well corresponds to a local low energy state (equilibrium). At each instant of deformation, the ball is excited to the apex, leading to non-equilibrium; after structural relaxation, the ball retreats to the bottom of the nearest valley, achieving local energy minimization, so that the network is in a meta-stable state. Theoretically, the real profile of the locally high or low energy state is continuous because of the numerous conformations available in the network. Moreover, each energy well should be extremely narrow. To visualize and simplify the idea for further discussion, the well-shaped discontinuous energy states are illustrated in Fig. 4a.

Figure 4a also shows how the energy is stored and how the shape is fixed during the programming process. Microscopically, the heat absorption enhances the motion of electrons and reduces the electron cloud density. Consequently, the deformation can be applied more easily and higher energy level can be achieved. When the temperature drops while maintaining the programming strain, the electrons localize to the associated atoms and this meta-stable conformation or structure of the network is frozen by the amplified energy well (the dotted green line in Fig. 4a). CSBs will locate at the bottom of the newly formed cold energy well. Because the depth of the energy well is enlarged, the CSBs are difficult to jump out of the cold well without a sufficient energy input. Therefore, the temporary shape is fixed. When the temperature is lower than the glass TZ, the bonds are not easily rotatable due to the lack in free space. Although the stretched bonds, which contain enthalpy, try to return the network to their original configuration after cooling and unloading, their energy is not sufficient to overcome the energy barriers formed by the surrounding neighbors. Hence, the enthalpy is stored in the stretched bonds.

Figure 4b shows the shape recovery process. For the free shape recovery, the cold energy well (the dotted green line) gradually gains energy and switches back to the hot energy well (the solid blue line) when the programmed network is reheated. Once a critical temperature is achived, here the onset point of the glass TZ, some bonds become rotatable. The CSBs are gradually lifted from the bottom of the well. The stretched bonds will attempt to contract and release their enthalpy by rotatable bonds into the whole continuous network. With further increase in temperature (energy input), the CSBs are lifted to the edge of this energy well by the stretched bond. If the absorbed energy of CSBs is greater than the energy barrier of the energy well and the network is not constrained externally, the CSBs can overcome the energy barrier and plunge back to the lower energy well. Eventually, CSBs will stabilize at the ground energy state. Macroscopically, the network restores the permanent shape, suggesting completion of the free shape recovery.

The stress recovery can also be discussed based on this energy well model. If the network is confined, the CSBs will stay at the edge of the last energy well (the deepest blue energy well) formed at the end of programing in Fig. 4a and generate the recovery stress. This recovery stress can be separated into two parts: the thermal stress and the memorized stress. The thermal stress is generated by the more strenuous movement of electrons in space. This drives the green colored energy well (cold) back to the blue colored energy well (hot) in Fig. 4b. The memorized stress can be further separated into two categories. The first category is generated by the micro-Brownian motion which is related to the entropy. The second category is generated by the retreat of bond length which is enthalpy related. During the reheating, in the glassy state, the thermal stress plays a major role. Once the temperature comes to the onset point of the glass transition zone, the memorized stress starts to release. For entropy, it generates recovery stress by micro-Brownian motion; for enthalpy, the bond length shortening applies forces to rotatable bonds, and accelerates the velocity of micro-Brownian motion to even higher energy level. The increased velocity, or kinetic energy, will transfer to the boundary of the specimen contacting the test machine, to produce the impact force or recovery stress, similar to gas motion in a container. In the energy well model, the stored stress highly depends on the depth of the final energy well (deepest blue well). The deeper the energy well, the more the energy can be stored and the higher the recovery stress is.

In summary, the energy and recovery stress in the rigid thermoset network can be stored by bond rotation and bond length change during programming, primarily by enthalpy increases. The stored energy or stress is locked by the valley of the cold energy well after programming. Reheating excites the CSBs jumping out of the energy well, and rolling down the energy hill, leading to either shape recovery, if no constraint is applied, or recovery stress, if constraint is applied and CSBs will stay at the edge of the final cold energy well. The value of the recovery stress and the energy stored by deformation is highly related to the depth of the final cold energy well formed at the end of programming. To enhance the recovery stress, enthalpy storage in terms of bond length changes is critical. Therefore, steric hindrance or interaction between the molecular segments need to be strengthened; see details in Supplementary Discussions. This will drive more energy storage in enthalpy form and reduce the relaxation during recovery, achieving higher recovery stress and energy output. Some approaches such as choosing monomers with high steric hindrance, using nano- or micro-fillers, employing double or multiple networks, molecules with not-easy-to-rotate structural element, etc., can be used; see discussion on some potential systems in Supplementary Methods.

## Methods

### Polymer synthesis

**Fig. 5:**
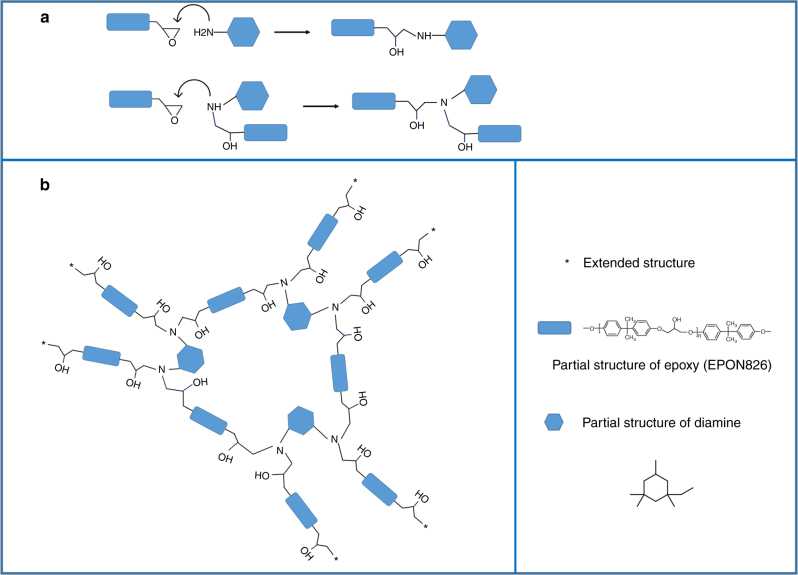
The possible reaction pathway for the EPON–IPD network. **a** Presents how one amino group reacts with an epoxy group. **b** Shows the network formed by nine EPON 826 and three IPD molecules. The stars indicate the extension of the rest of the network

Commercially available epoxy (EPON 826, DuPont, USA) and a rigid IPD, named as 5-amino-1,3,3-trimethylcyclohexanemethylamine (Sigma-Aldrich, USA) are selected as the two components of the thermoset network. Each 100 g EPON 826 was reacted with 23.2 g IPD to balance the stoichiometry. The reagents were mixed by a mechanical mixer for 2 min at room temperature, and then were placed into a rectangle Teflon mold. The air bubbles were extracted by vacuum at room temperature. After 1 h curing under 150 °C, a thermoset network was obtained. The potential reaction pathway is schematically shown in Fig. 5.

### DSC test

The DSC test was performed by DSC 4000 (PerkinElmer) for the investigation of the thermal behavior of the synthesized polymer network and the enthalpy release for programed sample. The temperature scan was conducted as following steps: equilibrate at 30°C for 3 min, heat to 170 °C, equilibrate at 170 °C for 3 min, cool down to 30 °C, and equilibrate at 30 °C for 3 min. Then the heating and cooling cycle is repeated. All heating and cooling rates were controlled as 10 °C/min.

### Dynamic mechanical analysis and thermal expansion test

The thermomechanical property of the synthesized polymer network was analyzed by a TA Instruments Q800 Dynamic Mechanical Analyzer. Using the multi-frequency mode, the three-point bending test was carried out with fixed displacement. The temperature was scanned at a rate of 10 °C/min. The thermal expansion behavior was also measured by the dynamic mechanical analysis (DMA) under the controlled force mode. The fixture was changed to the tensile clamps. The cyclic temperature was scanned from −25 to 180 °C.

### Programming and free shape recovery test

The sample was prepared into a cuboid and compressed by the mechanical testing system (MTS) QTEST 150 machine for 40% of strain at 170 °C. After the sample was cooled down to room temperature and unloading, it was placed back into the oven and was heated up to 170 °C to trigger the free shape recovery.

### Fully constrained stress recovery

The fully constrained recovery stress was tested by the specimens programmed by 45% compressive strain. The test was conducted by the MTS QTEST 150 machine for 8 h. Before placing the programmed sample into the oven, the inside environment of the oven has been stabilized at 170 °C for 1 h.

### Relationship between recovery stress and recovery strain

A fully constrained recovery stress test for samples programmed by 45% strain was used to obtain one boundary point in the recovery stress–recovery strain curve, here zero recovery strain. The value of the recovery stress was measured after the stress was stabilized for 1.5 h at 170 °C. Another boundary point is the free shape recovery test, here zero recovery stress. The samples were allowed to recovery free of constraint in the oven at 170 °C for half an hour. For other points in the recovery stress–recovery strain curve, the clamp of the MTS machine was positioned to allow 2.5%, 7.5%, 12.5%, 17.5%, 22.5%, and 32.5% recovery strains, respectively. All the tests were conducted at 170 °C for 30–40 min to obtain stabilized recovery stress. The exact recovery time was determined by the variation of the stress. When the change of the recovery stress was <0.01 MPa in 10 min, the value was taken and the test was stopped. The whole process was repeated for three different samples.

### Relaxation behavior at different temperature zones

The relaxation test was conducted at four different temperatures, which were 120, 155, 170, and 175 °C. All samples were compressed to 40% strain, and then the deformation was maintained to let the stress relaxation occur. All relaxation data were normalized by the peak stress, *σ*_0_, at the end of compression.

### Stepwise iso-strain compression-relaxation test

The sample was equilibrated in rubbery state, which was 175 °C, before compression. In each step, 2% compressive strain was applied, and then relaxation was allowed for 4 minutes. The sample was compressed for a total of 44% of strain. This test was conducted by the MTS QTEST 150 machine with an assembled oven controlled by a Eurotherm Controller (Thermodynamic Engineering Inc. Camarillo, CA).

### Raman spectroscopy

The measurements for the samples programmed by different strains were performed by LABRAM integrated Raman spectroscopy system manufactured by Johin Yvon Horiba. The 1 mW He–Ne Laser was used as the excitation probe and the wavelength was 632.81 nm. Both focusing and collecting the backscattered light were carried out by a 10× objective lens. The chemical shift was scanned from 800 to 1300 cm^−1^.

### X-ray spectroscopy

NEXAFS was conducted. The C 1*s* K-edge spectrum was collected and used for the analysis of carbon involved bonds. The first peak was identified as the C 1*s* → π* (C = C) peak at 285.4 eV by polystyrene. The spectrum collection was carried out by the GEOL 7900 X-ray absorption spectrometer associated with the low energy beamline from the synchrotron located at the Center for Advanced Microstructures and Devices (CAMD), Baton Rouge. The ground polymer powder was mounted on the copper tape as the testing sample. The compressed polymer network by different strains was milled by sandpaper gently in a −20 °C environment to reduce the heat produced by friction.

### Data availability

All other data are available from the authors upon reasonable request.

## Electronic supplementary material

Supplementary Information
