# Supplementary material for: High enthalpy storage thermoset network with giant stress and energy output in rubbery state
Source: Nat Commun. 2018 Feb 13;9:642. doi: 10.1038/s41467-018-03094-2 (PMC5811442; doi:10.1038/s41467-018-03094-2)
Supplement: Supplementary file 1 — Supplementary Information [file 41467_2018_3094_MOESM1_ESM.pdf]

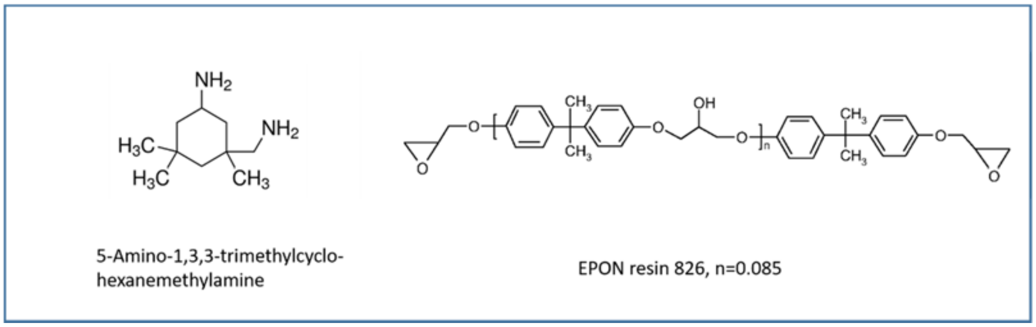

Supplementary Figure 1. Molecular structure of the reactants.

The isophorone diamine (IPD) with high steric hindrance is shown in the structure on the left-hand side. The rigid epoxy, which is EPON 826, is illustrated on the right-hand side.

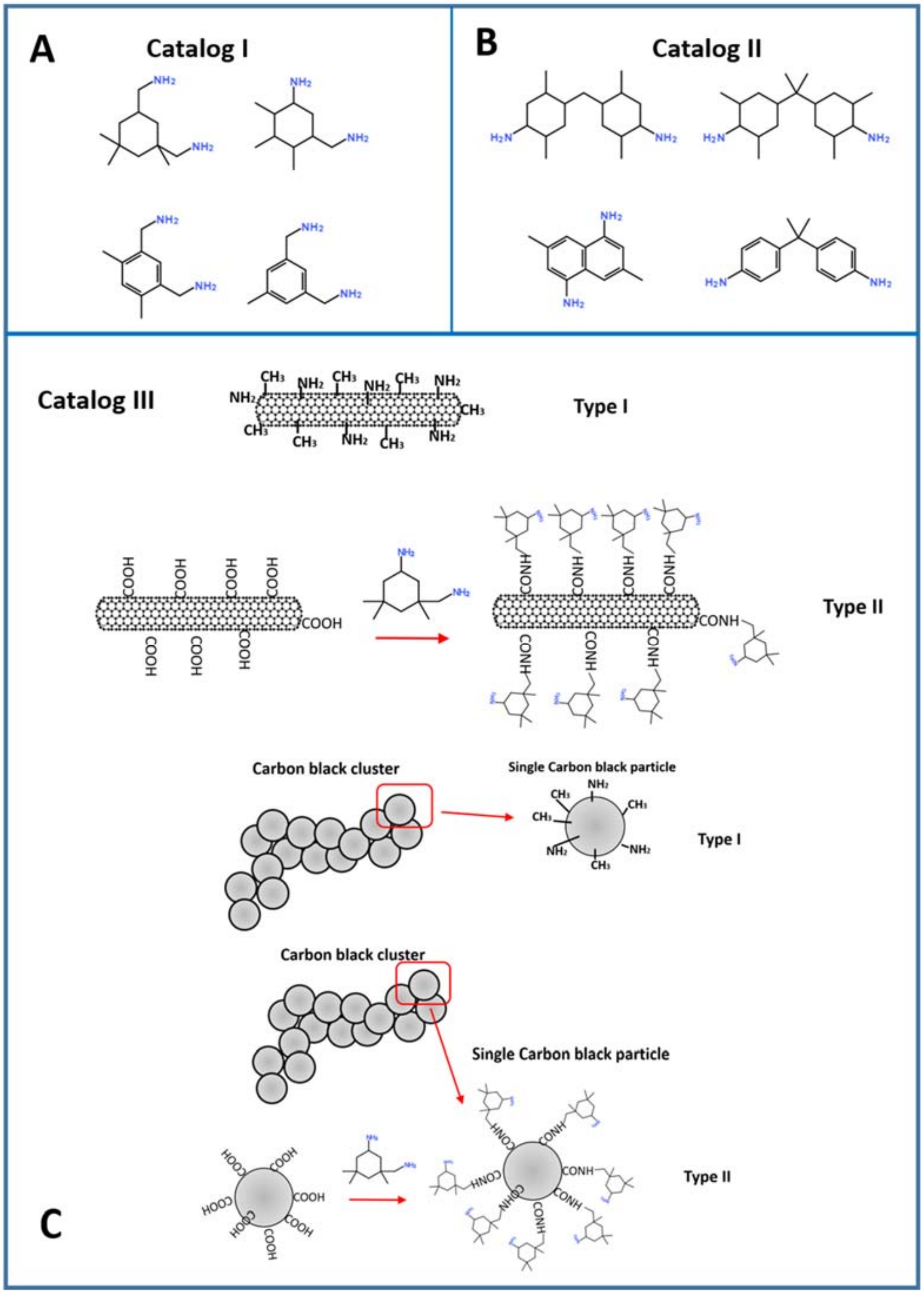

Supplementary Figure 2. Potential amide molecules for enthalpy storage thermoset polymer. (A) The chemicals in the catalog I are the diamines with mono-cyclic structure attached with groups that can supply hindrance. (B) The catalog II has poly-cyclic or heterocyclic structure. (C) The catalog III has carbon nanotube (CNT) or carbon black serve as the rigid center.

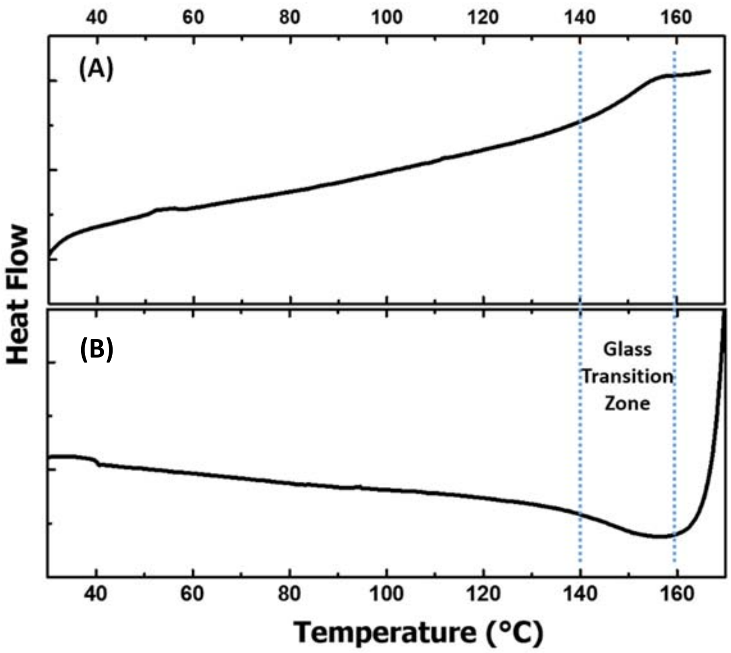

Supplementary Figure 3. DSC data profile for synthesized EPON-IPD polymer network.
(A) Represents heating curve and (B) represents cooling. The glass transition zone is identified between 140°C and 160 °C.

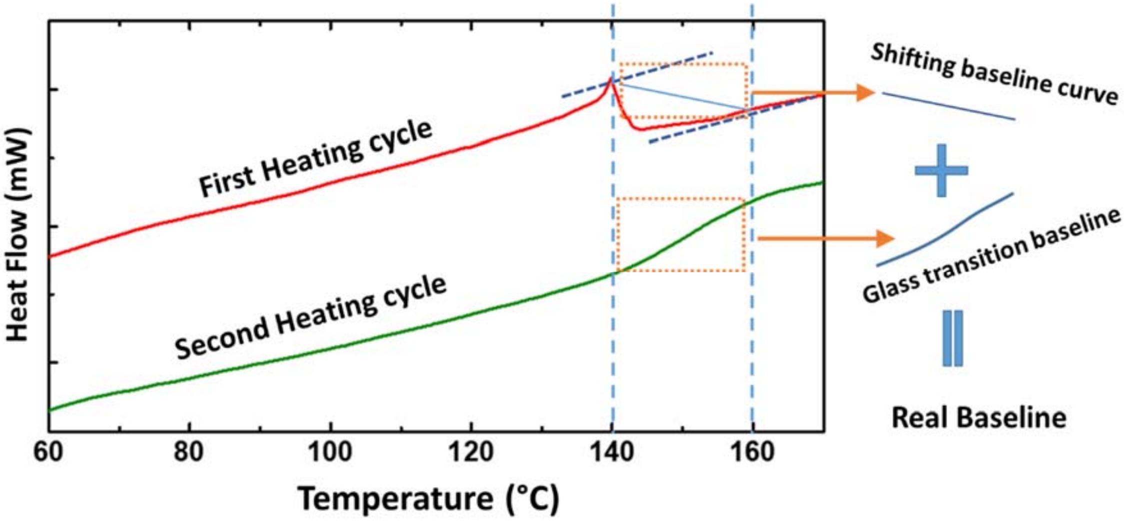

Supplementary Figure 4. The first and the second heat flow curve during heating.

The sample was programed with 40% pre-strain. The baseline of the heat release can be separated into two portions which are shifting baseline curve and the glass transition baseline.

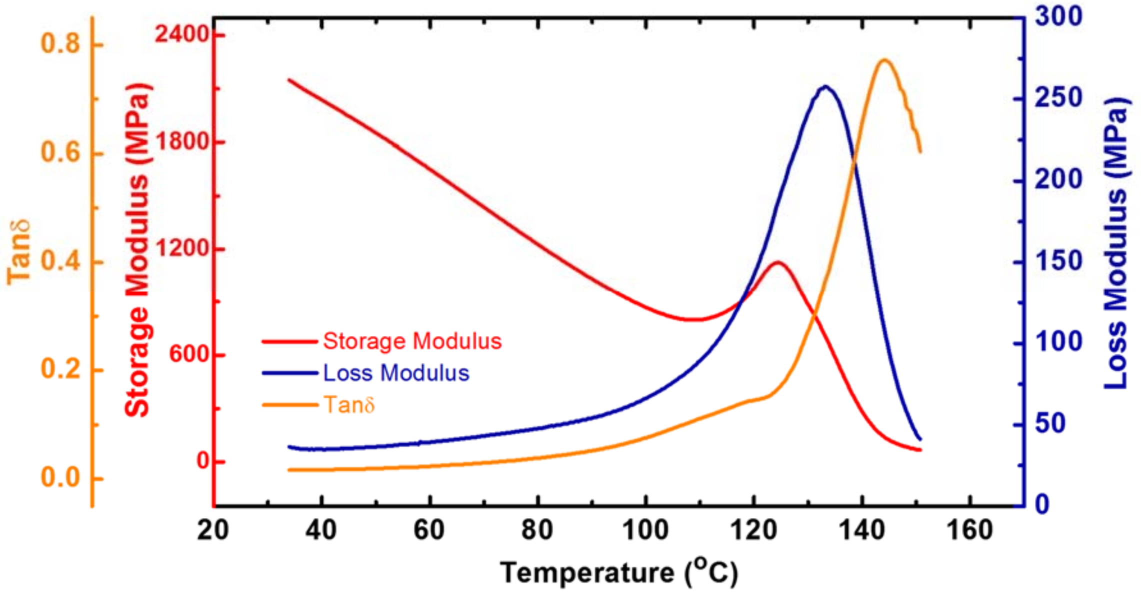

Supplementary Figure 5. The dynamic mechanical analysis profile.

Storage modulus, loss modulus and tan δ against temperature were scanned from room temperature to 150°C. Based on the peak of tan δ, the glass transition temperature is between 140°C and 150°C, which is slightly lower than the result from DSC. Discrepancy between DSC and DMA measurements has been common. Instead of several MPa for most entropy driven thermoset SMPs at temperature approaching the end of the glass transition region, which is a requirement for good shape recovery, the storage modulus of our polymer network is about 65 MPa at 150°C.

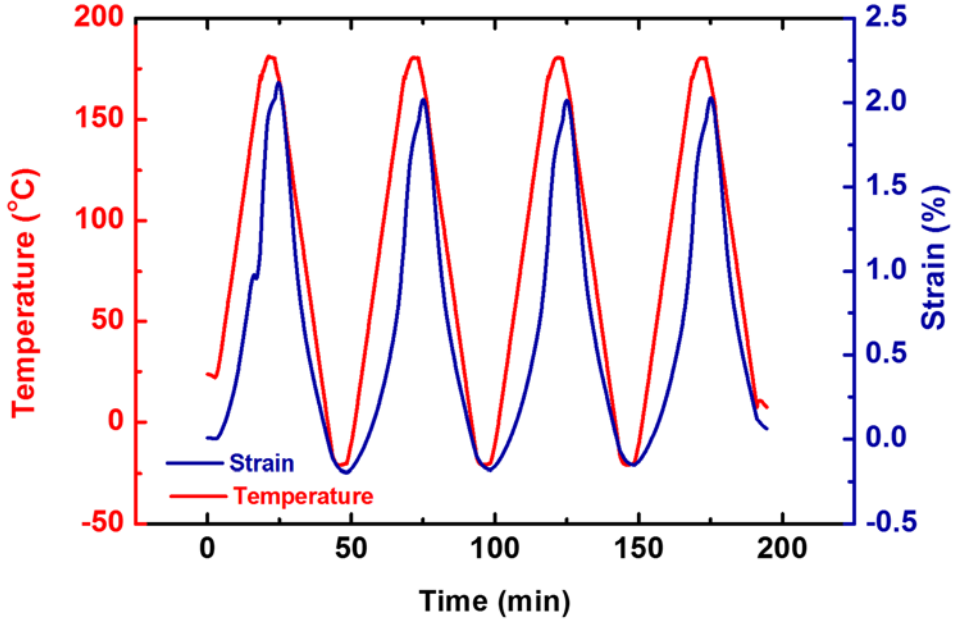

Supplementary Figure 6. The thermal expansion test performed by DMA.

The average coefficient of thermal expansion, which is equal to the strain during heating dividend by the corresponding temperature increment, is found to be 1.25× 10-4 °C-1 for the EPON-IPD polymer network. The serval rounds of heating and cooling cycles lead to almost the same test results.

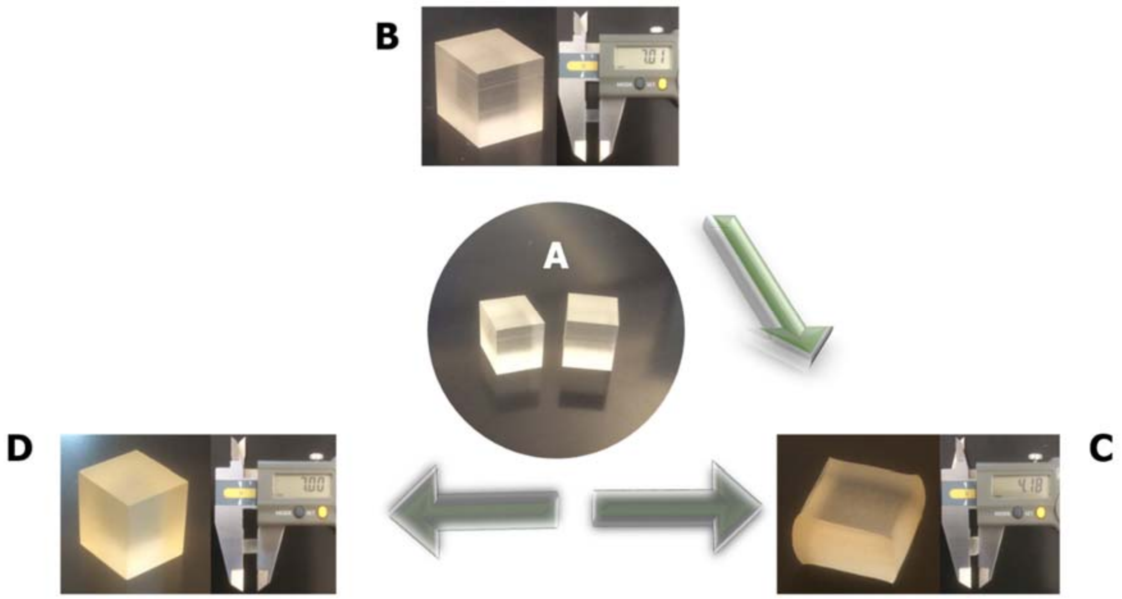

Supplementary Figure 7. The prepared samples and the free shape recovery test.

(A) The cut and milled cuboid samples. (B) The sample before the compression programming, which shows that the side length of the cuboid sample is 7.01 mm. (C) The sample after programming, which is compressed by 40% strain, and the height of the cuboid sample is 4.18 mm, which translates to a shape fixity ratio of about 100%. (D) The sample after the free shape recovery, almost fully restoring the original permanent shape (the side length becomes 7.00 mm after free shape recovery as compared to original length of 7.01 mm). This suggests that the high enthalpy storage network has excellent shape memory ability.

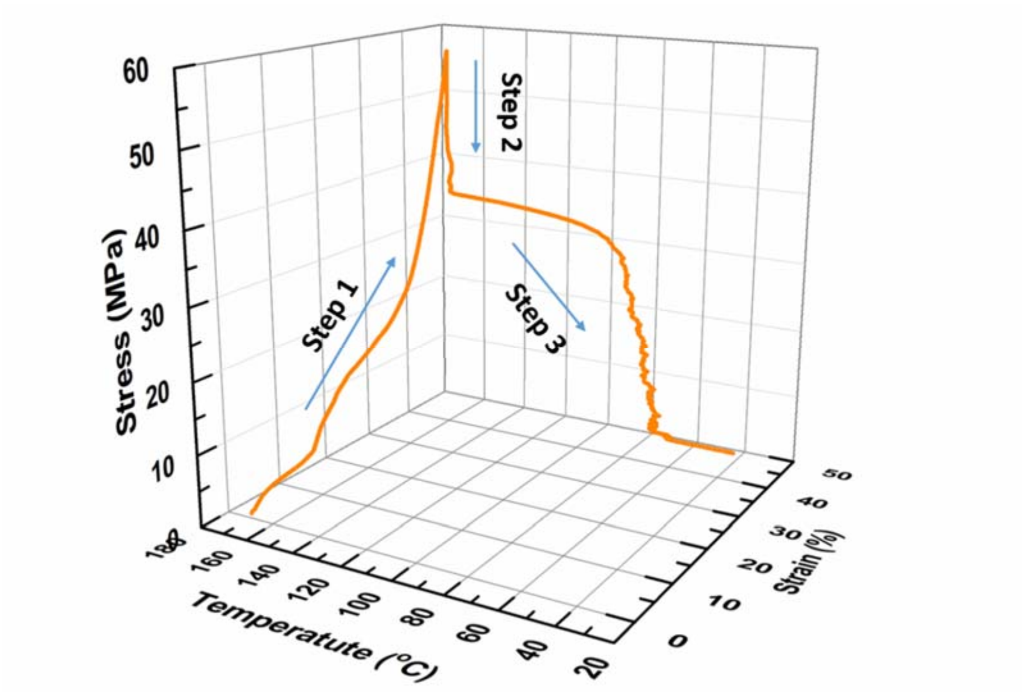

Supplementary Figure 8. Compression programming test.

It consists of compression at 170 °C (step 1), stress relaxation at 170°C (step 2) and cooling and unloading process (step 3).

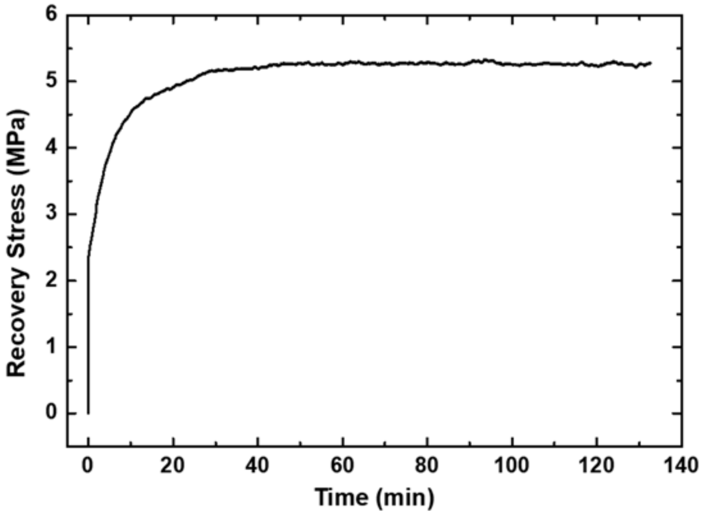

Supplementary Figure 9. Recovery stress test after tension programming.

The EPON-IPD specimen was tension programed by 10% tensile strain. The recovery stress increases with time and stabilizes at about 40 minutes.

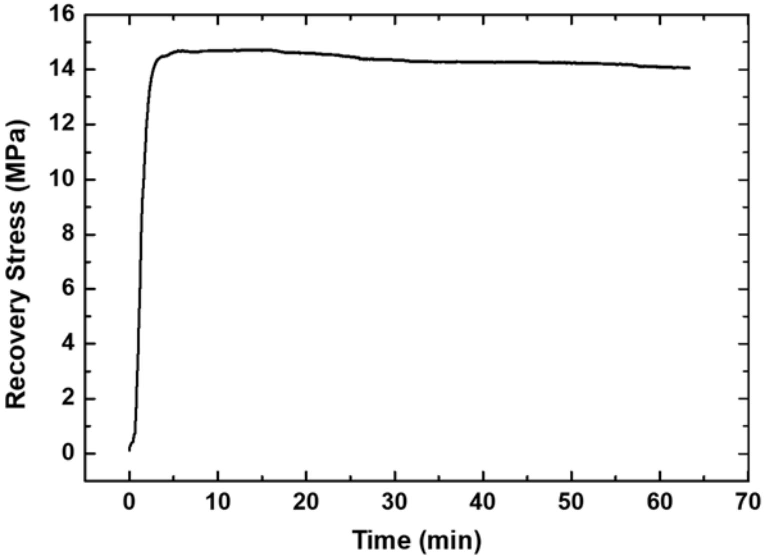

Supplementary Figure 10. Compressive recovery stress test.

The specimen was compression programed at 150°C (within the glass transition region) by 45% pre-strain. The recovery stress test was conducted at 170 °C (in rubbery state). The peak recovery stress is about 15MPa and the stable recovery stress is about 14 MPa.

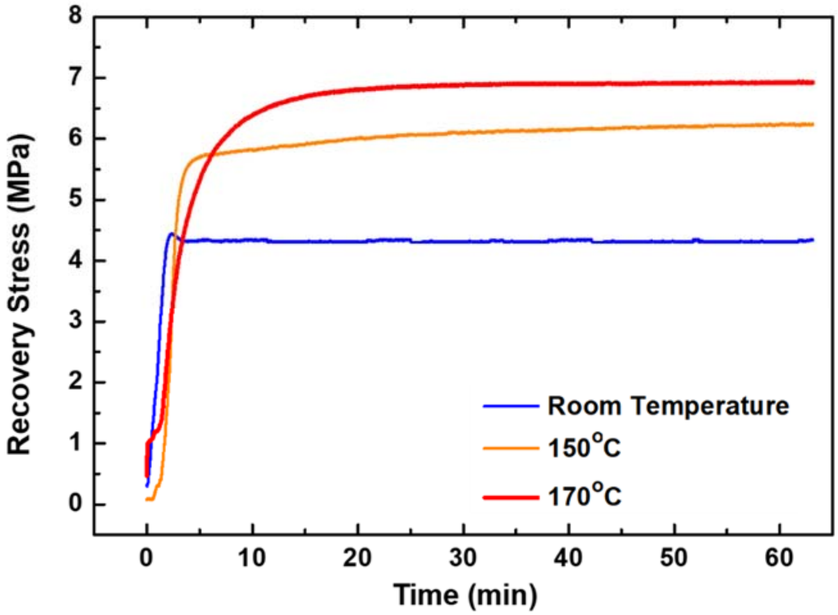

Supplementary Figure 11. Recovery stress test for samples with the same fixed strain.

The specimens were compression programed with a fixed strain of 32% at different temperatures. The recovery process was performed at 170 °C which is the rubbery state. Programming at glassy state leads to the lowest recovery stress, and programming at rubbery state results in the highest recovery stress.

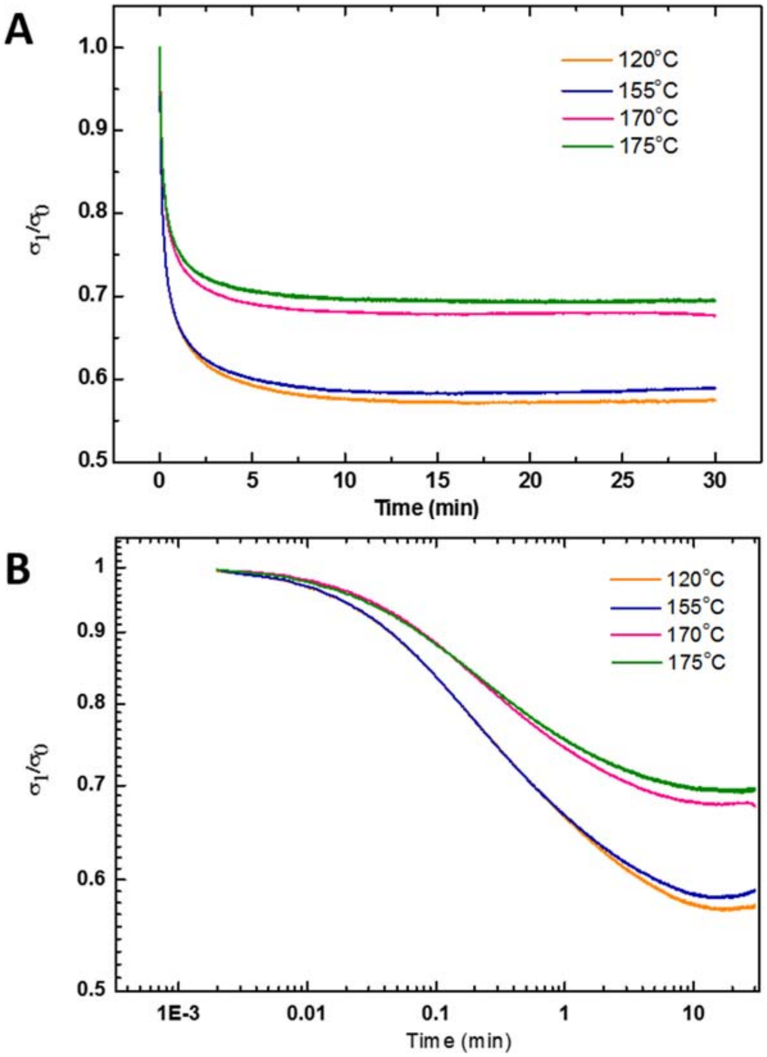

Supplementary Figure 12. The normalized stress relaxation profile at different temperatures.

The EPON-IPD polymer network was compressed to 40% strain, and then the deformation was maintained to let the stress relaxation occur. (A) Linear scale and (B) logarithmic scale. Stress relaxation is much larger at glassy state (120 °C) and glass transition zone (155 °C) than at rubbery state (170 °C and 175 °C).

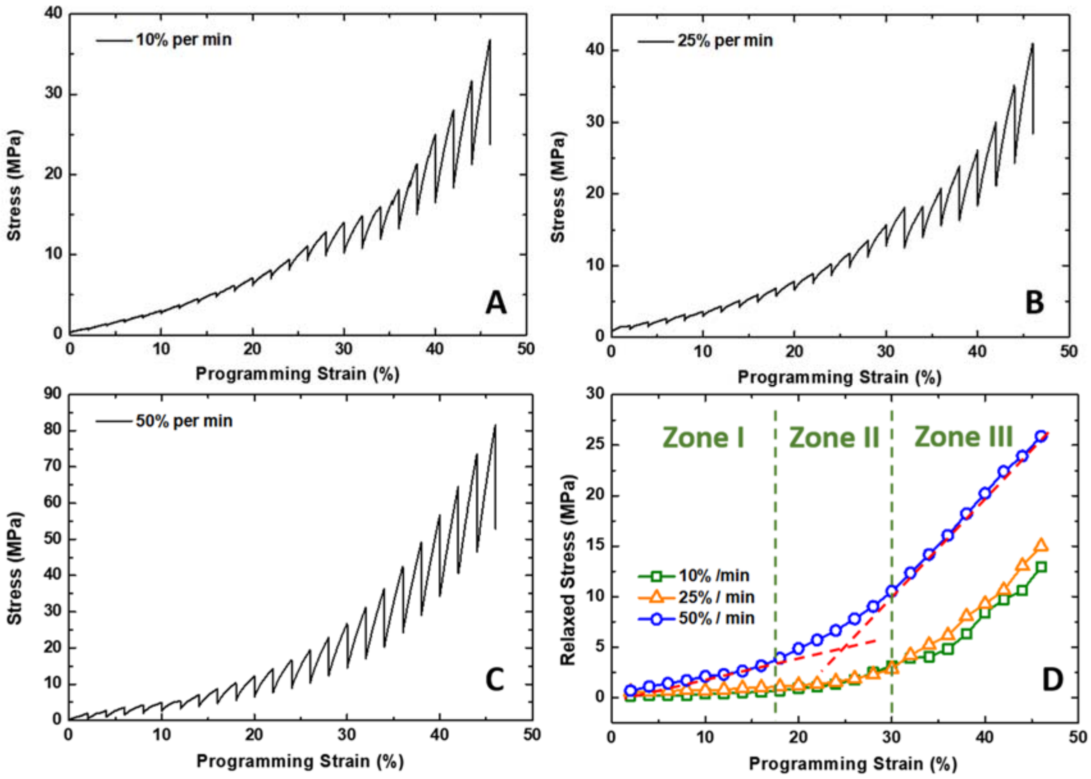

Supplementary Figure 13. Stepwise compressive programming and stress relaxation profile. Different programming strain rates were used. (A) The strain rate is 10% per minute. (B) The strain rate is 25% per minute. (C) The strain rate is 50% per minute. (D) The relaxed stress or stored stress for each step of the three different stepwise programming.

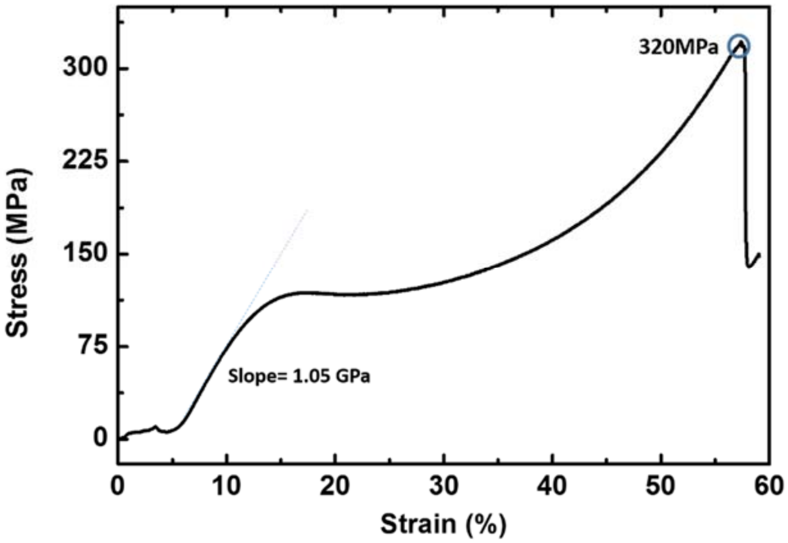

Supplementary Figure 14. The glassy state compression stress-strain curve.
The EPON-IPD network shows a typical linear elasticity, yielding, strain softening, plastic flow, strain hardening, and fracture at 320MPa.

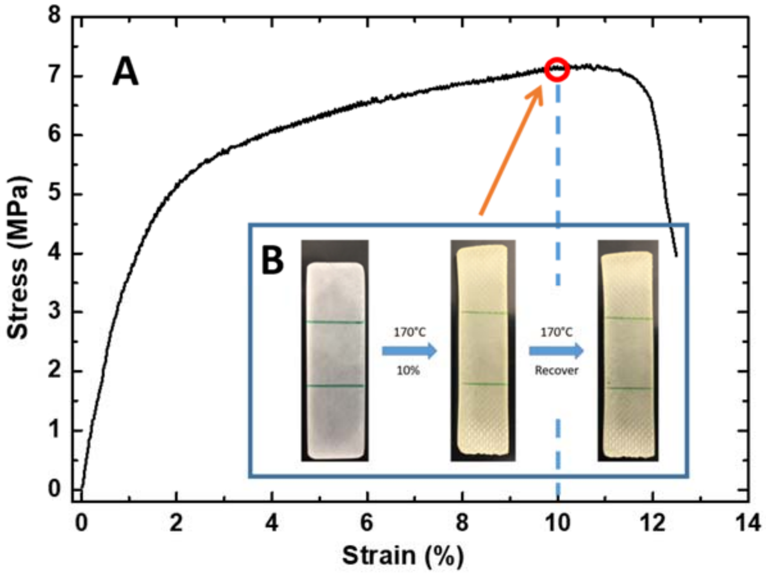

Supplementary Figure 15. Rubbery state tensile test.

(A) Relationship between stress and strain during the tensile test for a rectangular EPON-IPD specimen at 170 °C. (B) The images of a specimen before and after the tensile programming and the specimen after recovery with 10% programming strain.

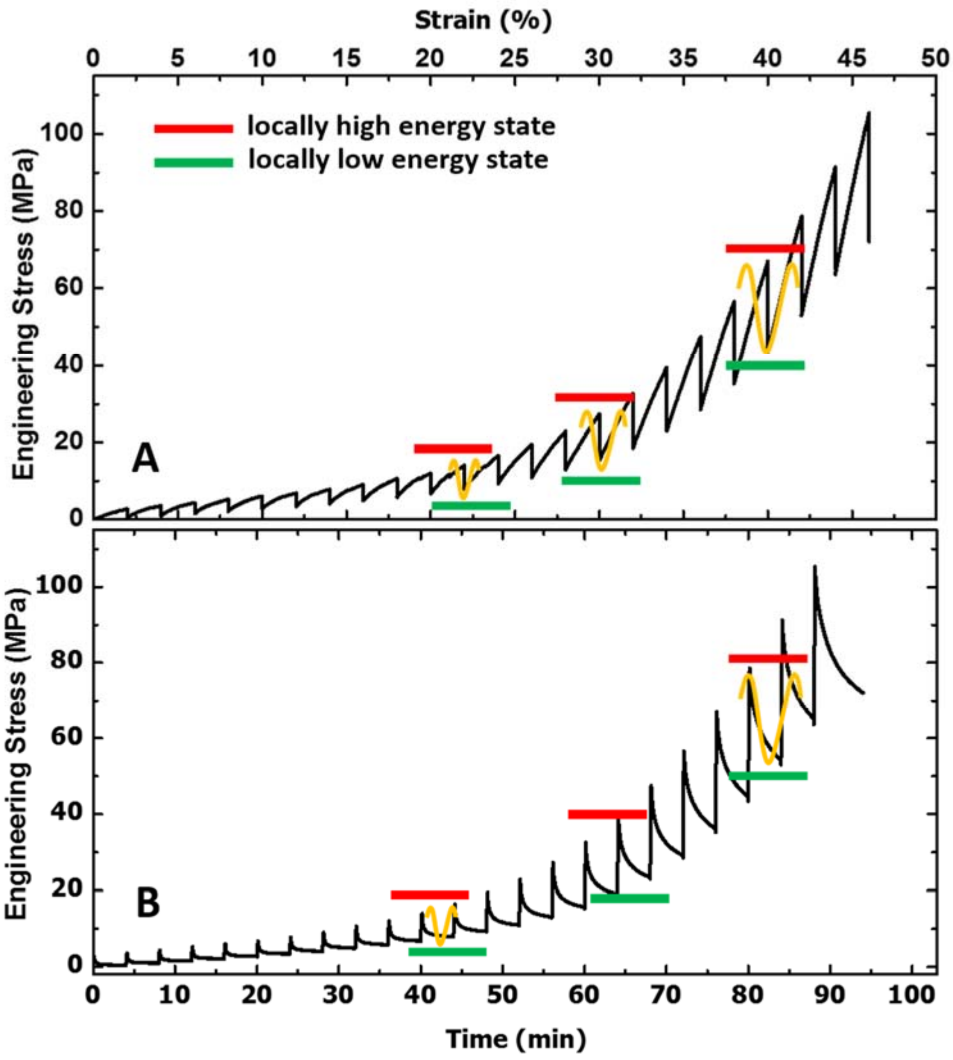

Supplementary Figure 16. Iso-strain compression and relaxation experiment.

The engineering stress is against (A) strain and (B) time. This curves clearly show the multiple energy wells along an ascending energy hill, i.e., deformation brings the network to a higher energy state, and relaxation brings the network back to a local lower energy state.

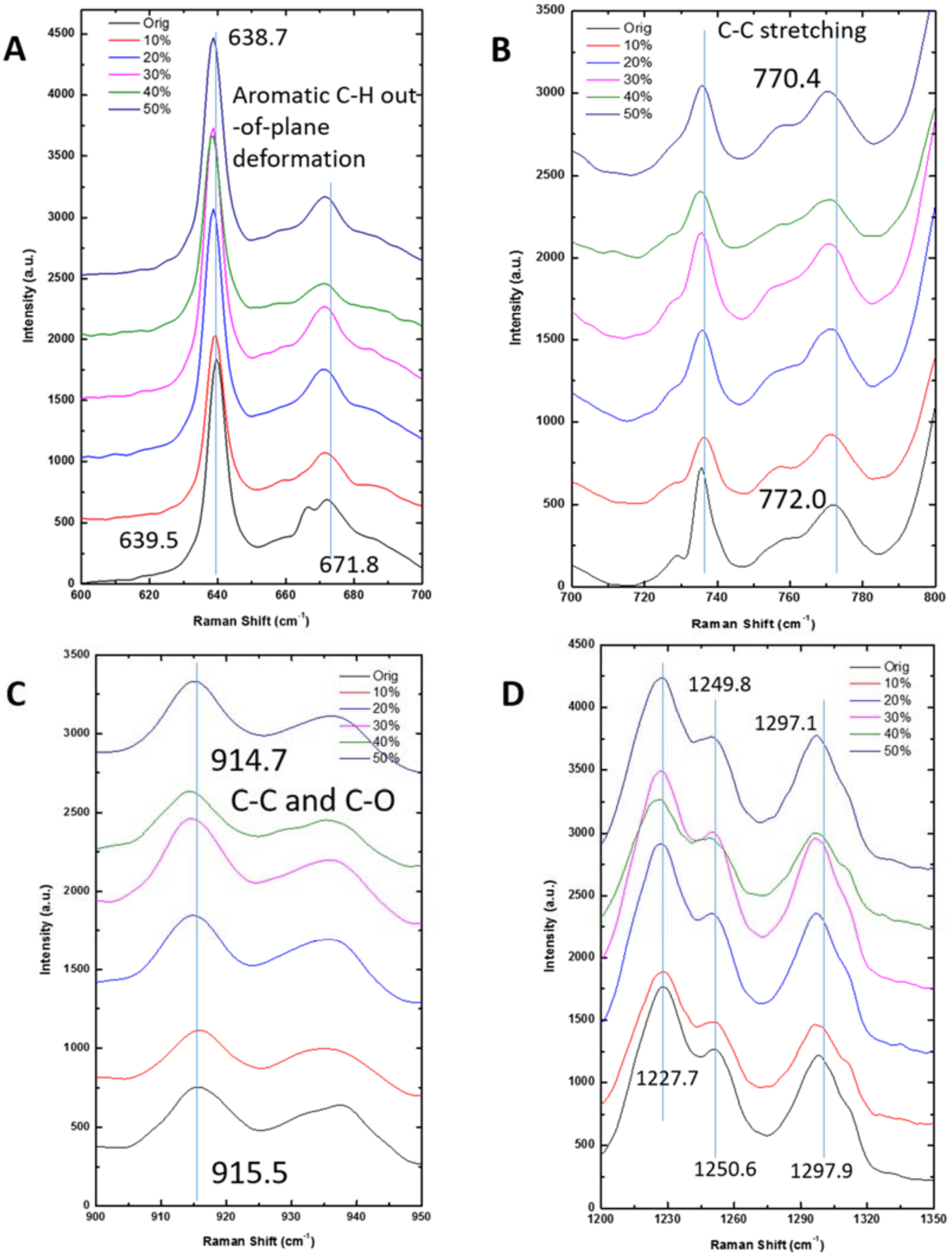

Supplementary Figure 17. The bond length change confirmed by Raman spectroscopy.

(A) Peaks for aromatic C-H out-of-plane deformation. (B) Peaks for C-C stretching. (C) Peaks for C-C or C-O stretching. (D) C-O stretching and phenolic C_4-O_2 stretching (1227.7 cm^{-1}); C-O-C stretching of the epoxy group (1250.6 to 1249.8 cm^{-1}); and C-O stretching (ether groups) and C-C stretching (1297.9 to 1297.1 cm^{-1}). From this Raman Spectroscopy, no shift happens for the programmed sample with 10% pre-strain such as Fig. 17C, where the pre-strain locates in the LZ1

in Fig. 1C. Therefore, bond rotation or dihedral angle change is the only mechanism for the deformation. The peaks begin to shift towards lower frequency direction for the sample with 20% programming strain, which falls on the TZ in Fig. 1C, meaning that the bond length begins to be stretched. Therefore, bond enthalpy starts to increase. Larger shift occurs for samples programmed by 30%, 40%, and 45% pre-strains, indicating that the bond length is stretched more and more in LZ2 in Fig. 1C.

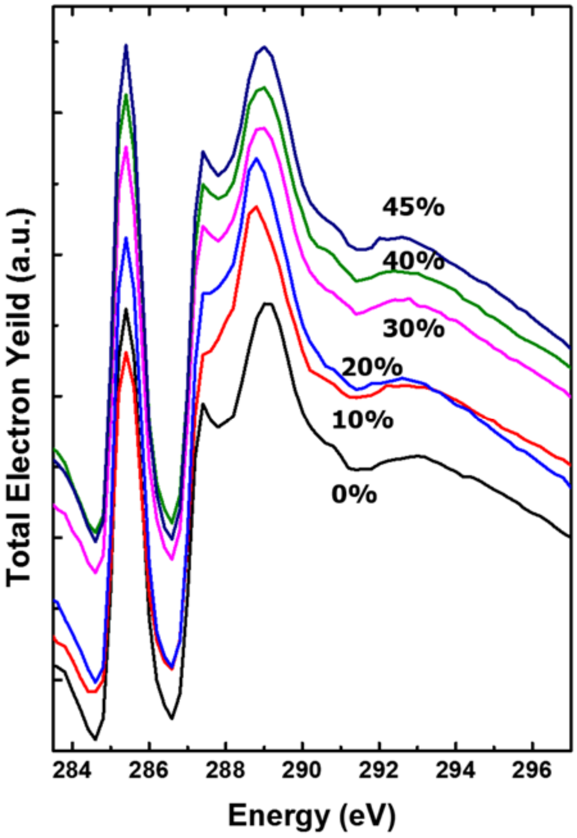

Supplementary Figure 18. The bond length change by X-ray spectroscopy.

Bond length change was confirmed by Near Edge X-ray Absorption Fine Structure Spectroscopy. The first peak located at the 285.4 eV is identified as the C 1s→ C=C* (ring) resonance, which is calibrated by polystyrene. The second and the third peaks located at 287.4 eV and 289.0 eV are peaks associated with the C-H bond in the ring. The area used in the study is the wide peak located in the energy higher than 291eV. The carbon associated single bonds are the resonance for peaks such as C-C, C-O or C-N bond. It is seen that there is no shift between the 10% programmed sample and the control sample without programming. Therefore, the bond length of the carbon associated single bond does not change. With the increase in programming pre-strain, the peaks begin to shift towards lower energy direction, which proves that the bonds are stretched. Larger programming strain leads to larger shift in peaks, suggesting higher bond stretch, which is similar to the result by Raman Spectroscopy.

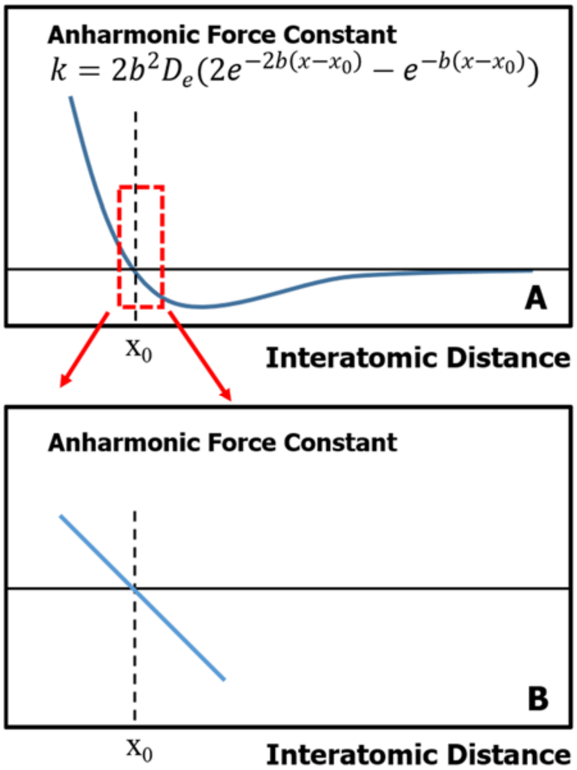

Supplementary Figure 19. The relationship between force constant of anharmonic oscillation. (A) full range of interatomic distance and (B) the small range around x_0. In a small variation around x_0, k decreases monotonically.

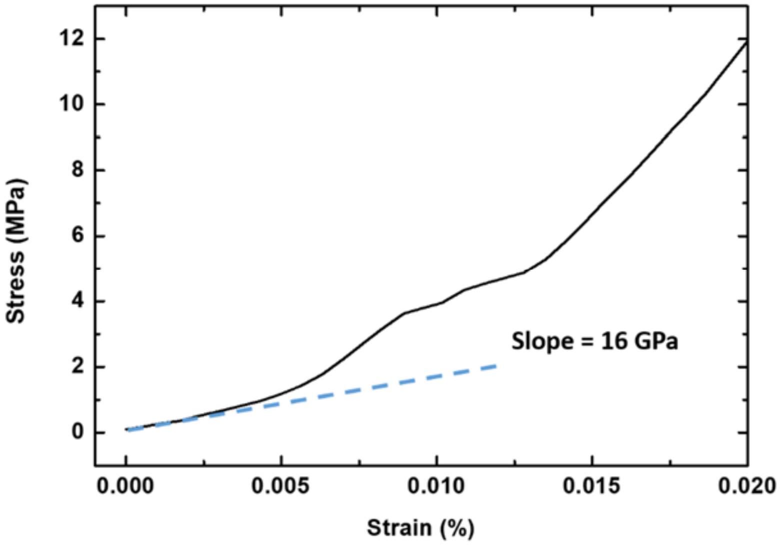

Supplementary Figure 20. Stress-strain relationship for programmed sample.

The sample was first programmed by 45% compression pre-strain. It was then compression tested again by applying a small compression strain.

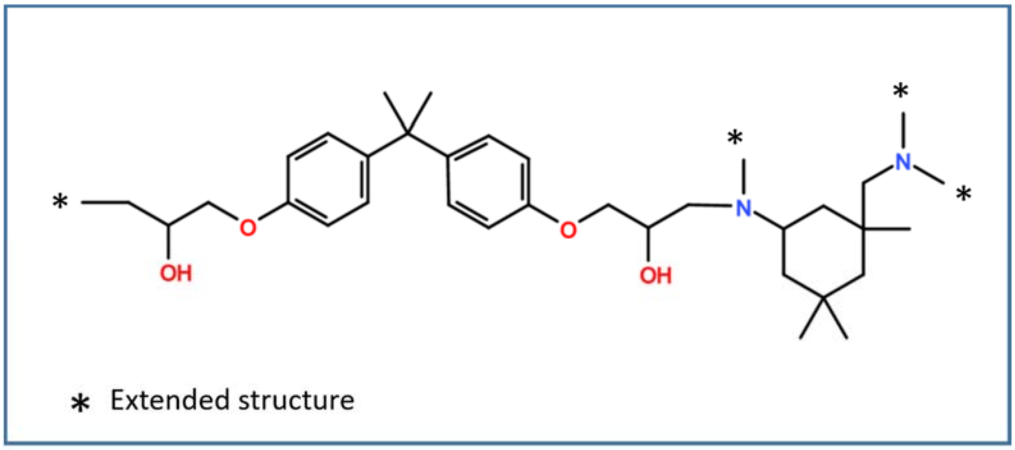

Supplementary Figure 21. The repeating structure of the EPON-IPD network.

The figure shows that one EPON 826 molecule, without the repeating unit of itself, reacts with one IPD molecule. When we count the number of different bonds for Supplementary Equation 11, one EPON 826 molecule and one half IPD molecule are counted because of the stoichiometry.

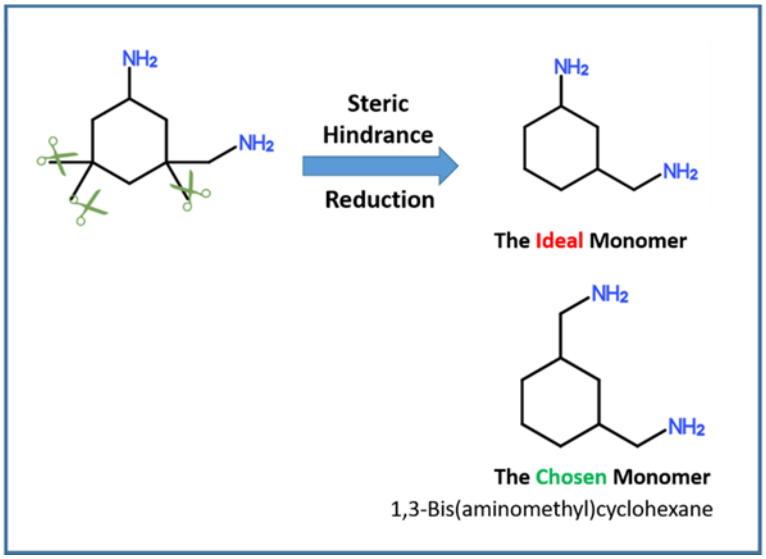

Supplementary Figure 22. The ideal and the chosen monomer (diamine).

The upper left-hand side shows the molecular structure of the IPD. The ideal monomer used for the investigation of the steric hindrance effect is the diamine without the three methyl groups as illustrated by scissor cutting. The monomer chosen in this study is the chemical shown on the bottom right-hand side, which is 1,3-Bis(aminomethyl)cyclohexane.

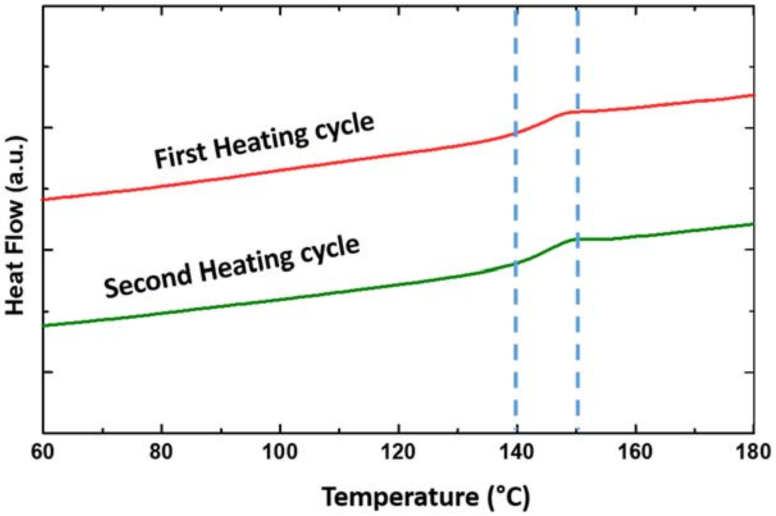

Supplementary Figure 23. The DSC data for the EPON-BACH thermoset network.

The sample was un-programmed. It consists of both the first and the second heating cycles. The glass transition region was identified by the two dotted vertical blue lines.

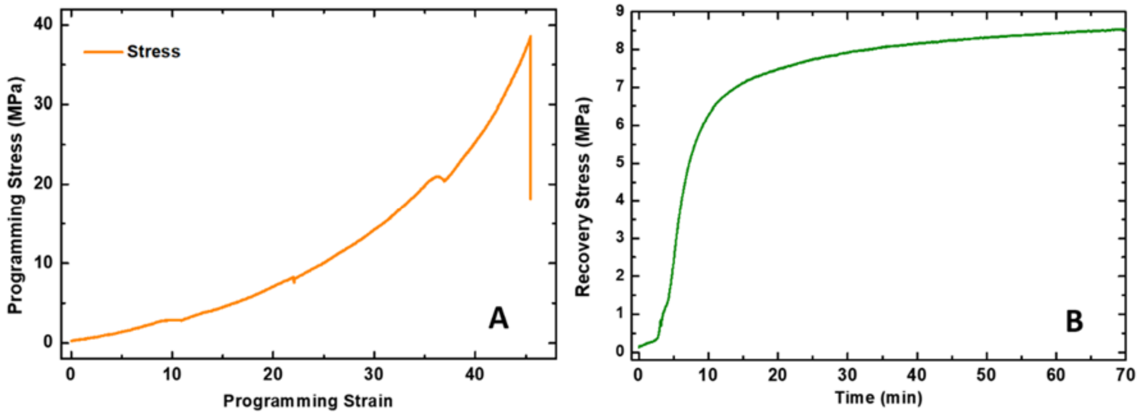

Supplementary Figure 24. Programming and recovery of the EPON-BACH polymer.

(A) The compressive programming stress with strain and (B) the compressive recovery stress evolution with time.

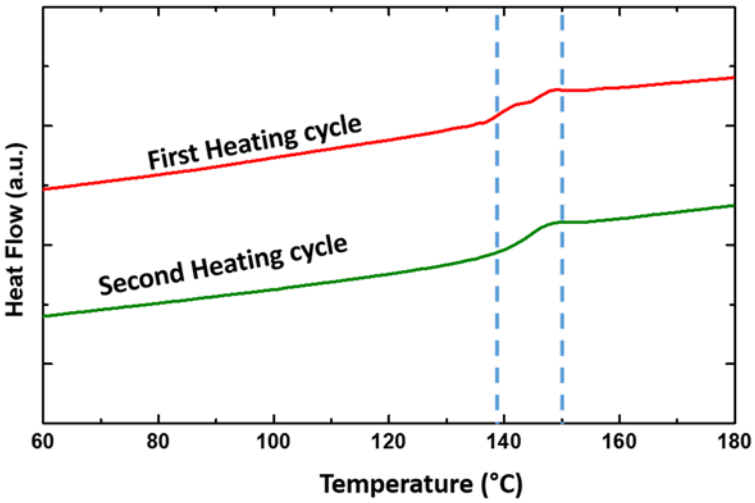

Supplementary Figure 25. The DSC data for the EPON-BACH network.

The specimen was compression programmed to 45% strain. It consists of both the first and the second heating cycles. The glass transition region was identified by the two dotted vertical blue lines.

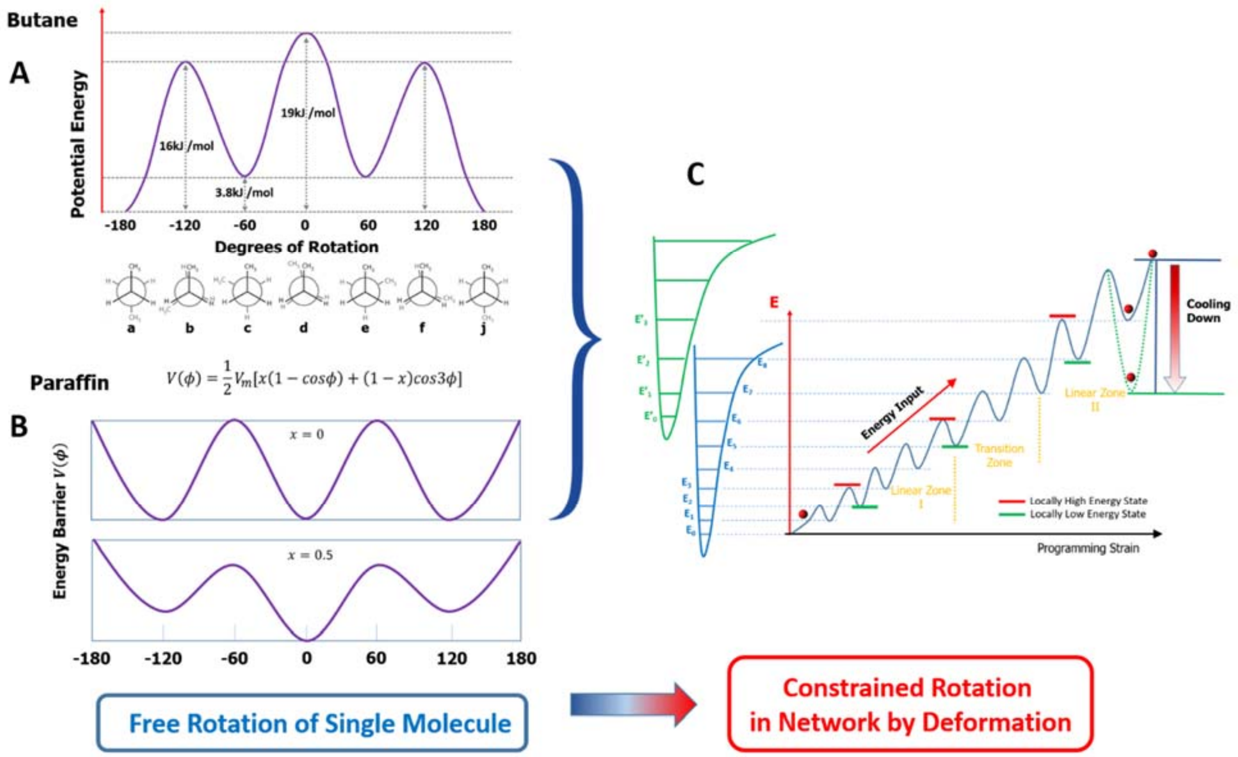

Supplementary Figure 26. The origin of the multiple energy well model.

(A) The relationship between potential energy and rotational angle for butane. (B) Two different cases for the energy barrier curve of paraffin based on Taylor's equation (*22*). (C) The multiple energy well model.

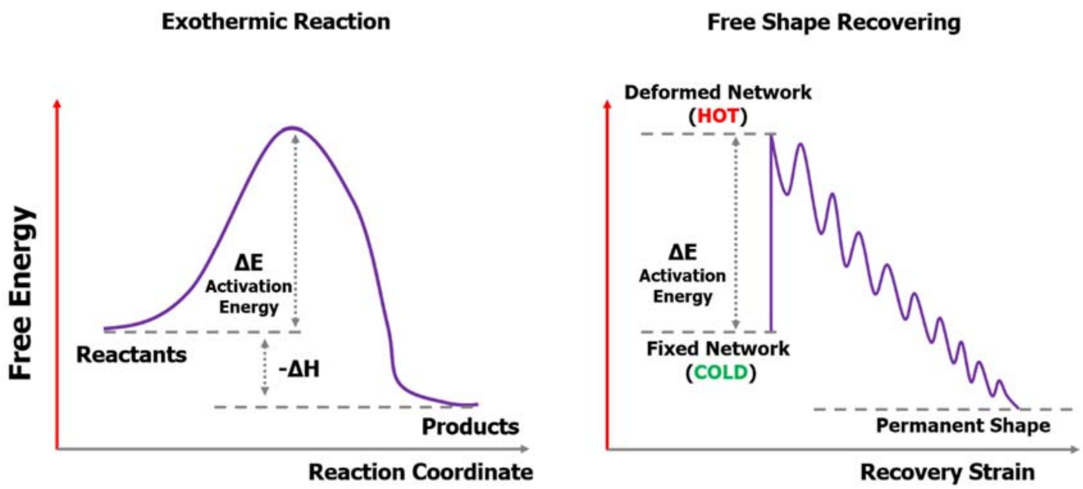

Supplementary Figure 27. The comparison of the exothermic reaction and free shape recovery. Both of them share the same mechanisms: while the internal energy is high in the non-equilibrium state, the systems need external energy input to overcome the energy barrier and trigger either the exothermic reaction or shape recovery.

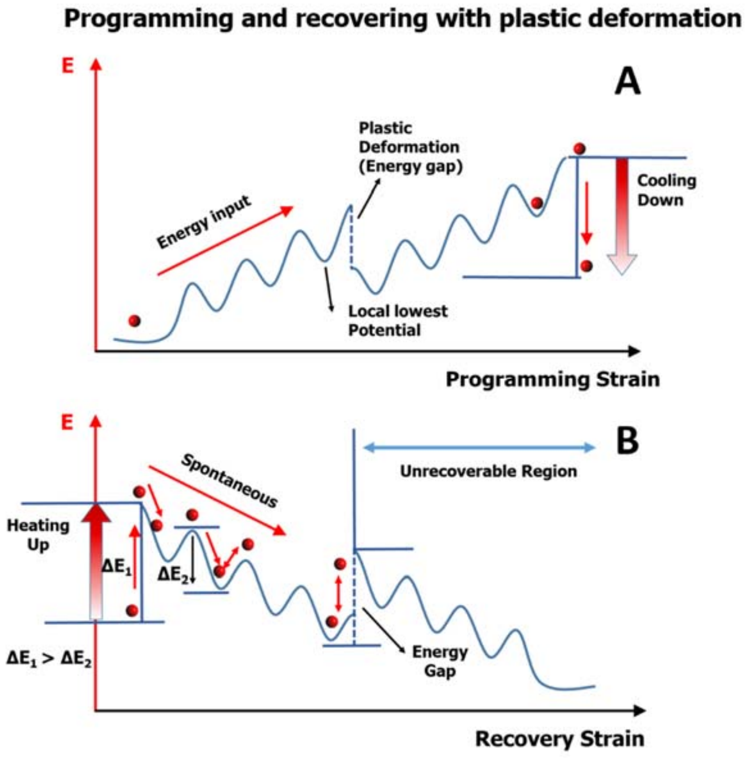

Supplementary Figure 28. Interpretation of plastic deformation by the multiple energy well model.

(A) The formation of energy gap during the programming. (B) The plastic deformation prevents the shape recovering by an energy gap.

Supplementary Table 1. Shape fixity ratios of the samples with different compression programming pre-strains.

Compressive Programming Pre-strain (%)	10	20	30	40	45
Shape Fixity ratio (%)	89.3 ± 5.3	87.9 ± 4.0	88.3 ± 3.2	84.9 ± 2.1	89.6 ± 2.2

Supplementary Table 2. The stress release and energy output in rubbery state for typical compression programmed pure thermoset shape memory polymers, recovery stress and energy output of a shape memory alloy, and energy output of typical elastically deformed metals.

Material Type	Recovery Stress (MPa)	Real Energy Output* (MJ/m^3)	Over-Estimated Energy Output** (MJ/m^3)	Pre-strain of Compression programming
EPON-IPD	17	2.12	3.82	45%
(meth)acrylate (*2*)	~1.5	N/A	~0.23	30%
Styrene based crosslinked SMP (*3*)	0.5	N/A	0.13	50%
Epoxy (TEMBO) (*4*)	0.12	N/A	0.05	80%
304 stainless steel (*5*)	N/A	0.10	N/A	1%
Ductile cast iron (*5*)	N/A	0.46	N/A	2.3%
Red brass (*5*)	N/A	0.83	N/A	4%
Shape memory alloy*** (*6*)	240	N/A	3.96	3.3%
PCL-2T-MA**** (*7*)	N/A	1.5	N/A	400%

*The real energy output is calculated by the area of the enclosed by the recovery stress-recovery strain curve for polymers. For metals, it is calculated by the elastic part of the area of stress-strain curve.

** The over-estimated energy output is calculated by the area of the right triangle determined by the fully constrained recovery stress and free shape recovery strain as the two vertexes of the right triangle.

***Tension programming (assuming modulus of elasticity of 85GPa, and 100% recovery ratio).

****Tension programming to 400%; the 1.5MJ/m^3 energy is storage energy.

Supplementary Table 3. The variation of the Raman shift of the different bonds due to programming to 45% strain.

Bond type	C-H	C-C	C-O (ester)	C-OH
ω_0 (cm^{-1})	639.5	772	915.5	1250.6
ω_{final} (cm^{-1})	638.7	770.4	914.7	1249.8
$\Delta\ \omega$ (cm^{-1})	0.8	1.6	0.8	0.8

Supplementary Methods

Synthesis of shape memory polymer

Due to the attractive potential as a mechanical actuator in future structural applications, a two-component thermoset network was chosen as the representative model polymer in this study. To uncover the relationship between the conformational, structural, energetical and mechanical characteristics at molecular level, a pure polymer network without reinforcing filler is an appropriate object. Commercially available epoxy (EPON 826, DuPont, USA) was used as the first component in the network. To enhance the enthalpy storage, intense steric hindrance is necessary to construct a stiff network. To prevent from losing stoichiometry during the reaction, a high reacting efficiency is required. Therefore, a rigid isophorone diamine (IPD), named as 5-Amino-1,3,3-trimethylcyclohexanemethylamine (Sigma-Aldrich, USA), was selected as the other component in this network. Because the functionality of epoxy is two while the functionality of diamine is four, each 100g EPON 826 was reacted with 23.2g IPD to balance the stoichiometry. The reagents were mixed by a mechanical mixer for two minutes at room temperature, and then were placed into a rectangle Teflon mold. The air bubbles were extracted by vacuum at room temperature. After one hour curing under 150°C, a thermoset network was obtained. The reagents are shown in Supplementary Fig. 1 and the reaction pathways are illustrated in Fig. 5, respectively.

Although not synthesized, we believe that the system has a certain synthetic flexibility. We think that diamines with rigid cyclic structure which provides the large steric hindrance, such as methyl groups, are potential molecules. They are possible to help the formed thermoset network to store the energy as enthalpy during programming. This catalog of potential molecules is listed in the Supplementary Fig. 2A. Moreover, the poly-cyclic and heterocyclic diamines with the groups that can provide steric hindrance are also considered as potential candidates as shown in Supplementary Fig. 2B as the second catalog. Other than cyclic, poly-cyclic or heterocyclic structure serving as the rigid structural center, caged structures, such as carbon black, carbon nanotube (CNT) or polyhedral oligomeric silsesquioxanes (POSS), with amino groups, are also possible candidates, once the rigid structural center is surrounded by groups that can provide enough steric hindrance (such as the type I structure shown in Supplementary Fig. 2C). We can also graft the IPD onto the surface of acidized carbon black or CNT (such as the type II shown in Supplementary Fig. 2C). These structures can be considered as the catalog three. In summary, if the amines have a rigid center, such as cyclic or caged structure, and grafting by these groups may

provide steric hindrance, which are the possible chemical structures for the enthalpy storage. Grafting EPON epoxy onto the surface of rigid center such as carbon black, CNT or some nanoparticles, may be another way of synthesizing this type of SMPs.

Differential scanning calorimetry test

The differential scanning calorimetry (DSC) test was performed by DSC 4000 (PerkinElmer) for the investigation of the thermal behavior of the synthesized polymer network and the enthalpy release for the programed sample. The glass transition range and glass transition temperature were determined by the second heating branch. The temperature scan was conducted as following steps: equilibrate at 30° for three minutes, heat to 170°C, equilibrate at 170°C for three minutes, cool down to 30°, and equilibrate at 30° for three minutes. Then the heating and cooling cycle is repeated from second step to fifth step. All heating and cooling rates were controlled as 10°C/min. The heating branches of each cycle for the synthesized polymer and programed polymer are plotted in the Fig. 2. The whole second cycle (heating and cooling) for the synthesized polymer is plotted in Supplementary Fig. 3.

The enthalpy calculation based on the DSC curve depends on the selection of the baseline and the endpoints. Unlike melting or crystallization, which have a clear peak and usually the associated software in the DSC machine can automatically calculate the enthalpy, glass transition (second order transition) is signified by a change in the base line, indicating a change in the heat capacity of the polymer. In order to determine the end points of the transition zone, the baselines before and after the transition are extrapolated; see the two dashed pink lines in the second heating cycle curve in Supplementary Fig. 4. Then the glass transition zone is determined as the temperature range at the intersection of the extrapolated baselines and the line extrapolated from the linear portion during the phase transition (dashed red line in the second heating cycle in Supplementary Fig. 4). The intersections of the dashed red line and dashed pink lines were treated as the end points of the glass transition region in this study.

Next, the baseline for the first order transition (enthalpy), i.e., the first heating cycle for the compression programmed specimen, was determined. We considered the natural physical process occurred during the first heating cycle of the programed sample. We assumed that the inverse peak shown in the first heating cycle in Supplementary Fig. 4 was a result of two competing physical processes. The first process was the normal glass transition, which absorbed heat, and the second process was the enthalpic energy release, which gave off heat. We also assumed that the evolution

of the heat flow due to the glass transition alone was almost the same between the first and second heating cycles (Normally, there is a little difference between the first and second heating cycles due to the processing history.). We further assumed that the actual baseline of the first heating cycle for the programmed sample was separated into two parts. The first part was the glass transition and the trend of the baseline was the same as the second heating cycle glass transition baseline shown in Supplementary Fig. 4. The heat flow due to the enthalpy energy release can cause the glass transition baseline shift to lower value. This shifting baseline curve shown in the Supplementary Fig. 4 was used as the correction for the glass transition baseline. In this study, we assumed that the shifting baseline curve was a straight line connecting the two end points in the glass transition region. The combination of the shifting baseline curve and the glass transition baseline was the real baseline for calculating the energy release. Based on this real baseline, the heat release between 140 °C and 150°C was calculated to be 2.85 J/g by integrating the heat flow curve. Based on the density of the EPON-IPD, the enthalpy release was found to be 3.25 MJ/m^3.

Dynamic mechanical analysis

The thermomechanical property of the synthesized polymer network was analyzed by a TA Instruments Q800 Dynamic Mechanical Analyzer (DMA). Using the multi-frequency mode, the three-point bending test was carried out with fixed displacement. The temperature was scanned at a rate of 10°C/min. The storage modulus, loss modulus and tanδ were recorded against temperature as shown in Supplementary Fig. 5.

The thermal expansion behavior was also measured by the DMA under the controlled force mode. The fixture was changed to the tensile clamps. The cyclic temperature was scanned from -25°C to 180°C. The obtained data are shown in Supplementary Fig. 6. From the calculation based on the data presented in Supplementary Fig. 6, the coefficient of thermal expansion is 1.25×10^{-4} $°C^{-1}$ for the EPON-IPD network.

Sample preparation and compression programming

A perfect alignment is a significant factor for the uniaxial compression test. Hence, the cured bulky polymer network was cut and then carefully milled into a cuboid. The tolerance for each pair of parallel surfaces was less than ten micrometers. The obtained cuboid samples are shown in Supplementary Fig.7A. All edges of the cuboid samples are between 6.5 mm and 7.5 mm.

Uniaxial compression programming was then conducted. Before this process, the oven, specimen and fixture have already been heated at 170°C for over an hour, to avoid the effect of thermal expansion. The compression process is shown in Supplementary Fig. 8. Step one represents the relationship between the stress and strain during the compressive deformation up to 45% strain at 170°C. After this, stress relaxation occurred in step two (Note: in the literatures, step 1 and step 2 are usually treated as one step. For clarity of presentation, we divided them into two steps). The step three shows the relationship between stress and temperature during the cooling process, while holding the strain constant. The air cooling process was performed by opening the door of the oven only. It is interesting to note that the unloading step, which is needed for a typical programming, is coupled with the cooling step. The load becomes zero at about 80 ^{o}C, due to thermal contraction of the specimen. The compression programming was completed when the temperature drops to room temperature.

In order to understand the shape fixity capabilities of the SMP, we have tested the shape fixity ratio of the polymer at different programming strains, which are listed in Supplementary Table 1. Both the mean and standard deviation are given. Each shape fixity ratio in the table is the average of the test results of three compression programming with the same programming strain. The compression programming was conducted at 170°C.

From the test results, the shape fixity ratio is quite stable for different programming pre-strains. These shape fixity ratios are regarded as very good values for such a stiff shape memory polymer. Lower programming pre-strain leads to a slightly higher deviation. This is a reasonable outcome due to the inherent instrument errors or resolutions.

Sample preparation and tension programming

During tensile programming, the specimen with a dimension of 50 mm × 14.5 mm × 5 mm was mounted onto one end of the grips of the mechanical test machine before the oven was equilibrated at 170°C for an hour. Then, the specimen was fixed by tightening the other end of the grips and tensile programming was executed. The specimen was stretched to 10% strain at 170°C. After holding for 10 minutes, the pre-stretched specimen was cooled down quickly to room temperature by spraying water onto the specimen while holding the programming strain constant. The load was then removed to fix the programed shape.

Supplementary Discussion

Free shape recovery test

Free shape recovery, as an important feature of shape memory polymers, is influenced by the deformation manner during the programming process. The polymer network in this study was an entirely continuous network. Permanent deformation rarely happens except for breaking the chemical bonds. Consequently, without defect and damage of the network, the free recovery should reproduce the permanent shape. To test this property, the sample, prepared in Supplementary Methods, was compressed by the Mechanical Testing System (MTS) QTEST 150 machine for 40% of strain at 170°C as shown in the digital photos in Supplementary Fig. 7B and 7C. After the sample was cooled down to room temperature and unloading, it was placed back into the oven and was heated up to 170°C to trigger the free shape recovery. The photo of the recovered sample is presented in Supplementary Fig. 7D. The free shape recovery ratio is 99.9%.

Fully constrained stress recovery test

The fully constrained recovery stress of a shape memory polymer indicates the potential as a mechanical actuator for future structural applications. Recovery stress is obtained by heating the network to above the glass transition temperature (in rubbery state), but without allowing any recovery strain. In order to obtain the stabilized recovery stress, the specimen was held at the recovery temperature for hours. To investigate this property, the fully constrained recovery stress test was conducted on specimens programmed by 45% compressive strain. The test was conducted by the MTS QTEST 150 machine for 8 hours, as shown in Fig. 1A in the main text. Before placing the programmed sample into the oven, the inside environment of the oven has been stabilized at 170°C for one hour.

For tension programmed specimens, the recovery stress evolution with time was determined following the same procedure as compression programmed specimens; as shown in Supplementary Fig. 9. From Supplementary Fig. 9, one can see that the specimen with 10% tensile pre-strain can produce 5.1 MPa stable recovery stress in the rubbery state. As shown in Supplementary Fig. 14, the tensile programming stress with 10% strain is about 7.0 MPa. We would say that with 7.0 MPa stress input, 5.1 MPa stress output (recovery stress) is reasonably high. However, because the tensile fracture strain of the polymer at 170 ºC is about 12%, we did not do tensile programming higher than 10%.

To consider the effect of programming temperature on the recovery stress, two types of additional compression programming were conducted. In one type, a compression programming at the glass transition region (150°C) has been conducted. The pre-strain is 45%, and the fixed strain is 41.8%, which is almost the same as the fixed strain by programming at rubbery state (170°C) with the same pre-strain 45%. The similar fixed strain makes the comparison meaningful.

In the second type of programming, three specimens were programed into the same fixed compressive strain which was 32% at different temperatures (20°C (glassy state), 150 ^{o}C (glass transition zone), and 170 ^{o}C (rubbery state)). All the programmed specimens, then, were recovered at 170°C under the fully constrained conditions.

From Supplementary Fig. 10, the peak recovery stress is about 15MPa and the stable recovery stress is about 14 MPa. Both the peak value and the stable value are lower than 17MPa, which is the stable recovery stress produced by the specimen programed in the rubbery state. This is an unusual phenomenon for shape memory polymers (SMPs). For entropy driven SMPs, the recovery stress is usually higher when the programming temperature lowers, i.e., glassy state programming has higher recovery stress than programming at glass transition zone, and the least is programming in the rubbery state. This can be understood due to the temperature memory effect, i.e., the recovery temperature is lower if the programming temperature is lower. At lower recovery temperature, the stiffness of the SMPs is higher, leading to higher fully constrained recovery stress.

For the enthalpy driven shape memory EPON-IPON network, it stores energy primarily through the enthalpy increase due to the change in bond length. Therefore, how much enthalpy is stored or how many bonds are stretched during programming determine the recovery stress produced in the rubbery state. As discussed in the main body of the paper, the bonds can be changed only when they are rotated to a very high energy level. Therefore, if some regions (segments) are not soft enough to rotate, most bonds located in the segments are not stretchable. This means that the ability for enthalpy storage is not fully taking effect. At higher temperatures, bond rotation is more likely, and thus enthalpy can be increased through bond stretch. In conclusion, for this enthalpy driven SMP, programming in rubbery state leads to higher recovery stress than that in glass transition zone, which can be further validated by Supplementary Fig. 11.

From Supplementary Fig. 11, it is clear that the higher programming temperature can produce higher recovery stress. This is a proof of the argument that, for this enthalpy driven SMP, higher programming temperature leads to higher recovery stress. It is interesting to note that, for

this SMP, temperature memory effect still exists. The specimen programmed at lower temperature recovers at slightly lower temperature. As mentioned previously, for entropy driven SMPs, this may lead to higher recovery stress for specimens programmed at lower temperature. For this enthalpy driven SMP, although this effect still exists, programming at lower temperature still leads to lower recovery stress. This is an evidence of the enthalpic dominance in this SMP system.

Relationship between recovery stress and recovery strain

A significant advantage for shape memory polymers, as compared to shape memory alloys (SMAs) or ceramics, is their large recovery strain. While SMAs have a very high fully constrained recovery stress, may be in hundreds of MPa, their free recovery strain is very small, may be less than 10%. Eventually, SMPs may output comparable energy against SMAs (*1*). For shape memory materials, fully constrained recovery stress and free shape recovery strain are the two extreme cases of measuring their memory capability. If recovery strain is allowed, the recovery stress will be reduced. In many applications, stress recovery must be accompanied by strain recovery, such as using shape memory effect for closing wide-opened cracks in self-healing applications or as actuators. Therefore, it is highly desired that SMPs have high recovery stress with considerable recovery strain. Actually, the area generated by the recovery stress – recovery strain curve is a direct measurement of the energy output. To obtain the relationship between recovery stress – recovery strain, the recovery stress at different recovery strains is tested as follows. A fully constrained recovery stress test for samples programmed by 45% strain was used to obtain one boundary point in the recovery stress – recovery strain curve, here zero recovery strain. The value of the recovery stress was measured after the stress was stabilized for 1.5 h at 170°C. Another boundary point is the free shape recovery test, here zero recovery stress. The samples were allowed to recovery free of constraint in the oven at 170°C for half an hour. For other points in the recovery stress – recovery strain curve, the clamp of the MTS machine was positioned to allow 2.5%, 7.5%, 12.5%, 17.5%, 22.5%, and 32.5% recovery strains, respectively. All the tests were conducted at 170°C for 30 – 40 minutes to obtain stabilized recovery stress. The exact recovery time was determined by the variation of the stress. When the change of the recovery stress was less than 0.01MPa in 10 minutes, the value was taken and the test was stopped. The whole process was repeated for three different samples, and the averaged recovery stress with one standard deviation at different recovery strains is plotted in Fig 1B in the main text. From Fig. 1B, we can calculate the area enclosed by the recovery stress-recovery strain curve, which yields 2.12 MJ/m^3. This value

is comparable with some shape memory alloys (SMAs) (*1*), and is much higher than thermoset SMPs and typical metals reported in the literatures, suggesting good energy storage and output capability; see Supplementary Table 2.

Stress relaxation behavior at different temperature zones

At room temperature, the polymer network, which is in glassy and non-equilibrium state, will also relax to the equilibrium state although it will take a very long time. This circumstance is referred to as physical aging. At a high temperature, especially when it is close to the glass transition zone, the relaxation is accelerated significantly. Thus, to analyze the compression behavior during programming, the relaxation performance is needed. The stress relaxation test was conducted at four different temperatures, which were 120°C, 155°C, 170°C and 175°C. All samples were compressed to 40% strain, and then the deformation was maintained to let the stress relaxation occur. All relaxation data were normalized by the peak stress, σ_0, obtained at the end of compression; see Supplementary Fig. 12. Although the higher temperature softens the thermoset network more, the intense steric hindrance helps the electrons pack more tightly. Consequently, the network is stable at even higher temperatures and this is one of the reasons for the giant stress recovery at rubbery state.

Strain rate effect on stress storage

Due to the time-dependent behavior of the polymer network, loading rate should have an effect on the relaxation behavior. We have conducted the stepwise stress relaxation test with three strain rates: 0.1mm/mm/min, 0.25mm/mm/min, and 0.5mm/mm/min; see Supplementary Fig. 13 A, B, and C. As expected, the stress increases as the strain rate increases; and the relaxed stress, or stored stress, see Supplementary Fig. 13D, also increases. This is understandable because higher strain rate means shorter time for stress to relax. It is noted that, regardless of the strain rate, the three zones exist; see Supplementary Fig. 13D. This suggests that the stress is stored by both entropy and enthalpy. However, we do see that higher strain rates lead to higher residual stress or stored stress, suggesting higher recovery stress and energy output.

Mechanical behavior at room temperature

Most applications of SMPs require that they work at ambient temperature. Hence, the mechanical property at room temperature is important. SMP samples were compressed until fracture at room temperature by the MTS QTST 150 machine. The strain rate was 1mm/min. The test results are shown in Supplementary Fig. 14.

Tensile behavior at rubbery state

The tensile stress-strain behavior of the SMP was also investigated at rubbery state. The specimens were fabricated into a rectangular shape with a dimension 50 mm × 14.5 mm × 5mm. The strain is calculated by the gauge length of 15mm of the specimen, which is the length between the two marks as shown in Supplementary Fig. 15. The test temperature was 170ºC, and the strain rate was 0.03mm/mm/min. One can see that the polymer can only be stretched by about 12% strain before it fractures at 170ºC. The peak stress or tensile strength of the SMP is about 7.1 MPa. Therefore, when we tested the tensile recovery stress of the SMP, we selected 10% strain as the tensile programming pre-strain at 170°C.

Stepwise iso-strain compression-relaxation test.

Temperature, as a critical parameter affecting the mechanical properties of polymers, can be separated into different regions around the glass transition. When the temperature is lower than the glass transition zone, sufficient energy input is needed to render the coordinated segmental rotation to occur. Within the glass transition zone or at even higher temperatures, the bond rotation can happen at any strain because the thermal energy has already overcome the energy barrier for segmental bond rotation. Therefore, the deformation applied is an energy source to compel the polymer network into a non-equilibrium and locally high energy state. The relaxation will happen to stabilize the total energy towards a locally low energy state simultaneously. Thus, the characteristics of the relaxation is associated with the conformational and structural evolution during deformation. However, the relaxation reflected on the testing machine is always delayed because the relaxation is time dependent. Hence, to uncover the conformational and structural variation hidden during the deformation, a stepwise iso-strain compression-relaxation test was performed as follows. The sample was equilibrated in rubbery state, which was 175°C, before compression. In each step, two percent compressive strain was applied, and then relaxation was allowed for four minutes. The sample was compressed for a total of forty-four percent of strain. This test was conducted by the MTS QTEST 150 machine with an assembled oven controlled by a Eurotherm Controller (Thermodynamic Engineering Inc. Camarillo, CA). The stress against applied strain and temperature are plotted in the Supplementary Fig. 16.

Raman spectroscopy

Raman Spectroscopy, as a characterization method for the vibrational energy of chemical bond, is a very useful tool for revealing the variation of the bond length (*8,9*). In this study, bond

length is a significant parameter for enthalpy storage. After programming (rubbery state compression, cooling and unloading), a temporary configuration is fixed in the network. Whether or not the bond length has been changed can be observed by Raman Spectroscopy at room temperature. The measurements for the samples programmed by different strains were performed by LABRAM integrated Raman spectroscopy system manufactured by Johin Yvon Horiba. The 1mW He-Ne Laser was used as the excitation probe and the wavelength was 632.81 nm. Both focusing and collecting the backscattered light were carried out by a 10× objective lens. The Raman shift was scanned from 800 cm^{-1} to 1300 cm^{-1}. The shifting of peaks is labeled with the type of bond as shown in Supplementary Fig. 17. Both qualitative and semi-quantitative result can be obtained.

X-ray spectroscopy

To further confirm the change of bond length, the Near Edge X-ray Absorption Fine Structure (NEXAFS) Spectroscopy technique was also used. NEXAFS as a specific element related technique can resolve the electronic structure of molecule or molecular fragments (*10,11*). Carbon is the main element in the synthesized polymer network. Therefore, the C 1s K-edge spectrum was collected and used for the analysis of carbon involved bonds as shown in Supplementary Fig. 18. The first peak was identified as the C 1s→ π* (C=C) peak at 285.4 eV by polystyrene. The spectrum collection was carried out by the GEOL 7900 X-ray absorption spectrometer associated with the low energy beamline from the synchrotron located at the Center for Advanced Microstructures and Devices (CAMD), Baton Rouge. The grounded polymer powder was mounted on the copper tape as the testing sample. Subsequently, the sample was anisotropic and the shifting of the peak in the spectrum was due to the variation of the bond length only. The compressed polymer network by different strains was milled by sandpaper gently in a -20°C environment to reduce the heat produced by friction.

Enthalpy storage

The chemically cross-linked network in the rubbery state can be treated as a supramolecule. When the deformed subject is treated as an elastic body in rubbery state, the energy stored is described by the Mooney's equation (*12-14*):

$$W = C_1\left(\alpha_x^2 + \alpha_y^2 + \alpha_z^2 - 3\right) + C_2\left(\frac{1}{\alpha_x^2} + \frac{1}{\alpha_y^2} + \frac{1}{\alpha_y^2} - 3\right) \qquad \text{(Supplementary Equation 1)}$$

where C_1 and C_2 are constants, α_x , α_y and α_z are stretches in three-dimensional coordinate. For example, $\alpha_x = L_x/L_{x0}$ where L_x is the length after deformation in x direction and L_{x0} is the original length along the x axis. If the volume is assumed to be a constant, $\alpha_x\alpha_y\alpha_z = 1$. As a simplified case, let $\alpha_x = \alpha$, $\alpha_y = \alpha_z = 1/\alpha^{1/2}$, the retractive stress τ , given by $\frac{dW}{d\alpha}$, is:

$$\tau = 2C_1\left(\alpha - \frac{1}{\alpha^2}\right) + 2C_2\left(1 - \frac{1}{\alpha^3}\right) \qquad \text{(Supplementary Equation 2)}$$

If α is the stretch ratio in uniaxial test by a mechanical testing machine, the retractive stress can be used as the prediction of the deformation stress applied by loading. When α is greater than 1, the sample is under tensile test. On the other hand, if the sample is compressed, the value of α is less than one. In this case, the value of τ is negative, which represents that the retractive stress turns to compression.

The first term in the right-hand side of Supplementary Equation 2 is actually related to the change of conformational entropy. The change of the conformational entropy per volume (ΔS) is described by the following equation:

$$\Delta S = -\frac{\rho R}{2M_j}\left[\alpha^2 + \frac{2}{\alpha} - 3\right] \qquad \text{(Supplementary Equation 3)}$$

where ρ is density, R is the gas constant, and M_j is the molecular weight between closest crosslinking points or chain entanglements. The associated retractive or expansive stress (σ_s) is derived by $-d(T\Delta S)/d\alpha$ as the following equation:

$$\sigma_s = \frac{\rho RT}{M_j}\left[\alpha - \frac{1}{\alpha^2}\right] \qquad \text{(Supplementary Equation 4)}$$

Obviously, if let $2C_1 = \rho RT/M_j$, Supplementary Equation 4 becomes the first term on the right-hand side of Supplementary Equation 2; in other words, the first term on the right-hand side of Supplementary Equation 2 is indeed generated by entropy change.

Although the Supplementary Equation 2 can be used for many cases of polymer deformation in rubbery state, especially for rubbers, the second term on the right-hand side needs more understanding. The physical meaning of the constant C_2 in the second term of Supplementary Equation 2 is not fully understood. For most rubbers, the second term functions as a correction term because the result of the first term is not far away from the test result. However, for the

EPON-IPD network, if only the entropy term is used for the calculation of the retractive stress, i.e., using Supplementary Equation 4 alone, the retractive stress is calculated to be $\sigma_s = 20.5\ MPa$ when the following parameters are used: $\rho = 1.143 \times 10^{-3} g.mm^{-3}$, $R = 8.314\ J.mol^{-1}.K^{-1}$, $T = 170\ ℃ = 443K$, $M_j = 446.29\ g.mol^{-1}$ and $\alpha = 0.6$. This retractive stress value is much lower than the corresponding programming stress as shown in Fig. 1C, which is about 60 MPa. Therefore, entropy alone cannot capture the energy stored in the network. It is noted that, since the functionality of EPON is two and the functionality of IPD is four, it means that each EPON molecule is shared by two IPD molecules, but each IPD molecule is shared by four EPON molecules. Therefore, the weight of the repeating unit should be defined as one EPON molecule and half IPD molecule. This repeating unit can also serve as the chain between the cross-linking points and the molecular weight is 446.29g/mole.

Therefore, for programming strain up to 40%, mechanism other than entropy must be considered. From section of stress needed to change the bond length in Supplementary Discussions, we will find that the stress needed to stretch the bond is about 43.8 MPa. If we combine the entropy stress 20.5 MPa and the enthalpy stress 43.8 MPa, we obtain a total stress that needed to deform the sample is 64.3 MPa, which is very close to the programming stress of about 60 MPa. Therefore, for larger programming strain, enthalpy increase is indeed a way of storing energy. From the Supplementary Equation 2, the second term on the right-hand side is possibly dominant more than the first term because the value of α is less than 1 in compression programming. The $1/\alpha^3$ term is greater than $1/\alpha^2$. Therefore, the second term is likely related to enthalpy increase, or bond stretch.

From the analysis in the main body of the paper, the energy storage is still entropy dominant when the programming strain is less than 20%, which can be confirmed by Supplementary Equation 4. The calculated entropic stress is 2.5 MPa and 5.7 MPa for the 10% and 20% programmed sample. They are comparable with the programming stress in Fig. 1C, which are 4.1 MPa and 9.0 MPa, respectively. The sample with 10% programming strain only needs a slight correction by the second term of Supplementary Equation 2. The sample with 20% programing strain needs a little more correction by the second term in Supplementary Equation 2 because the bond length stretching enthalpy has already begun to take effect in the transition zone.

Recovery Stress

The energy storage mechanisms in the shape memory network can be further explained by the recovery stress. Let's first assume that the energy is stored by entropy only. The recovery stress at the maximum programming strain can be estimated by the following empirical equation for the change of entropy (*15*):

$$\Delta S_S = 5.2819\,(\varepsilon_{max})(\sigma_R) \qquad \text{(Supplementary Equation 5)}$$

and

$$\Delta S_s = 1.4011\,\frac{(tan\delta_{max}^2)(v_j)^{0.6613}}{\ln(v_j)} \qquad \text{(Supplementary Equation 6)}$$

where ΔS_S is the stored entropy, ε_{max} is the maximum programming strain and the σ_R is the associated recovery stress, $tan\delta$ is the ratio of loss modulus to storage modulus, and v_j is the cross-link density which equals to ρ/M_j as defined in equation Supplementary Equation 3 or Supplementary Equation 4. The constants in Supplementary Equation 5 and Supplementary Equation 6 were obtained by curve fitting. Plugging in Supplementary Equation 5 to Supplementary Equation 6, the empirical equation for σ_R can be derived as follow:

$$\sigma_R = \frac{1.4011(tan\delta_{max}^2)(v_j)^{0.6613}}{5.2819(\varepsilon_{max}) \times \ln(v_j)} \qquad \text{(Supplementary Equation 7)}$$

By using the same parameters applied to the section of enthalpy storage in the Supplementary Discussions and the value of $tan\delta$ being 0.77 (from the data in Supplementary Fig. 5), σ_R is calculated for the 45% programed sample, which is equal to 7.0 MPa. This value is much lower than the measured recovery stress shown in Fig. 1A and 1B in the main text. Therefore, entropy reduction alone fails to predict the test result. Enthalpy increase can explain the difference between the measured recovery stress (about 17 MPa) and the entropic recovery stress (7 MPa).

Stress needed to change the bond length

From the section of enthalpy storage in the Supplementary Discussions, energy storage mechanism other than entropy reduction must be considered to explain the difference between test results and model predictions. The vibrational energy associated with the chemical bond is an effective indicator for the change of the bond length such as carbon-carbon single bond. Raman spectroscopy, as the characterization technique analyzing the vibrational energy corresponding to the chemical bonds, is a powerful tool to determine the change of the bond length qualitatively. The semi-quantitative approximation can also be done by using the proportionality constant,

between the change of chemical bond shift and the stress needed to cause the bond shift. The detailed theoretical explanation is as follows.

The potential energy of chemical bond during the deformation is approximated by the Morse function (*16*) for anharmonic oscillation:

$$U_p = D_e(1 - e^{-b(x-x_0)})^2 \qquad \text{(Supplementary Equation 8)}$$

where U_p is the potential energy, D_e is the dissociation energy which is the energy needed to break the bond. Here b is a constant that equals to $\sqrt{k_e/2D_e}$, where k_e is the force constant at the minimum point of this function. The term $(x - x_0)$ is the change of interatomic distance.

The second derivative of Supplementary Equation 8 provides the force constant of the oscillation as following:

$$k = 2b^2 D_e(2e^{-2b(x-x_0)} - e^{-b(x-x_0)}) \qquad \text{(Supplementary Equation 9)}$$

According to Tashiro (*17*), the chemical shift or frequency (ν) is proportional to $\sqrt{k}$. From Supplementary Equation 9, in a small range around x_0, k decreases monotonically as shown in Supplementary Fig. 19. Therefore, when Δx is positive, $\Delta \nu$ is negative and the chemical bond is under stretching. To the opposite, when Δx is negative, the force constant increases, causing the frequency shift to higher values.

Based on Rretzlaff and Wool (*18*), the variation of frequency ($\Delta \nu$) is proportional to the applied stress. In our case, the change of the chemical bond shift in the Raman spectroscopy is observed without external loading, thus it is caused by the internal stress. This internal stress is also proportional to $\Delta \nu$.

The standard method to characterize the correlation between the Raman peak shift and the internal stress is the *in-situ* testing. The variation of the Raman shift should be observed during the deformation. The relationship between the peak shift and the external loading can be assumed as a linear fashion. During Raman test, the deformation is stepwise or very slow. Therefore, the internal stress is assumed the same as the external loading. Curve fitting may also be needed to estimate the precise coefficient. Based on this discussion, the EPON-IPD network also needs the coefficient for all the types of bonds. Without the *in-situ* Raman spectrometer associated with the mechanical deformation accessories, as a rough estimation, we turn to the equation proposed by Wei et al. (*19*), which links the internal stress, Raman shift, and modulus of elasticity of the materials:

$$\sigma_{bond} = \frac{E}{1-\nu} \times \frac{\Delta\omega}{\omega_0} \quad \text{(Supplementary Equation 10)}$$

where σ is the residual stress, E is the Young's modulus, ν is the Poisson's ratio, Δω is the variation of the Raman shift, and ω_0 is the reference Raman peak (original peak). The Poisson's ratio for the EPON-IPD is set as 0.48, which is an acceptable value for the nearly non-compressible thermoset polymer. The variation and the reference Raman peak can be obtained by the Raman spectrum. An additional parameter is the Young's modulus of the programed sample. Because the Raman spectrum was collected from the programed samples at room temperature, the Young's modulus with the same condition should be tested and utilized. Hence, the programed EPON-IPD sample with the 45% pre-strain is deformed with a very small strain as shown in Supplementary Fig. 20. The Young's modulus of the programed sample is estimated by the slope of the initial stress-strain curve, which is 16.0 GPa. The variation of the Raman shift for different types of bond due to the stretching is calculated and summarized in the Supplementary Table 3.

It is noted that Supplementary Equation 10 is based on one single type of bonds. In our SMP, it consists of several types of bonds; see Supplementary Table 3. Because the Young's modulus in Supplementary Equation 10 is for the entire network, we cannot use it to obtain the internal stress for each individual bond and then sum them up. A better way may be to use the rule-of-mixture's approach, which needs to consider the percentage of each type of bonds within the network. Therefore, Supplementary Equation 10 is revised to Supplementary Equation 11:

$$\sigma_{interal} = \frac{E}{1-\nu} \times \left(\frac{\Delta\omega_{CH}}{\omega_{CH}^{o}} \times \frac{N_{CH}}{N_{total}} + \frac{\Delta\omega_{CC}}{\omega_{CC}^{o}} \times \frac{N_{CC}}{N_{total}} + \frac{\Delta\omega_{CO-ester}}{\omega_{CO-ester}^{o}} \times \frac{N_{CO-ester}}{N_{total}} + \frac{\Delta\omega_{CO-Epoxy}}{\omega_{CO-Epoxy}^{o}} \times \frac{N_{CO-Epoxy}}{N_{total}}\right) \quad \text{(Supplementary Equation 11)}$$

where $\sigma_{internal}$ is the stored internal stress due to programming. *N* stands for the number of bonds and the subscript of *N* means the type of bond in a representative molecular unit (repeating unit). The subscript total is the sum of the number of bonds for all types of bonds within the repeating unit, i.e., $N_{total} = N_{CH} + N_{CC} + N_{CO-ester} + N_{CO-Epoxy}$.

Next, let us count the numbers for each type of bonds in the repeating structure. This percentage is the same for the whole network when we neglect the defects and end groups. For simplification, we also neglect the repeating unit in EPON 826 because only very low portion of the EPON has the repeating unit (8.5%). We count the number of bonds per Supplementary Fig. 21, which includes Aromatic C-H: 8; ester C-O: 4; C-OH: 2; and -C-C-: 6 + (4/2) = 8. All counts

are straightforward except for the number of carbon-carbon single bond. Firstly, there are 6 such bonds in EPON structure which are excluding the carbon connecting the benzene ring. There are four in the IPD which are excluding the carbon belonging to cyclic hexane. Because only half of the IPD needs to be counted, the four bonds is divided by two. Consequently, the total number of carbon-carbon single bonds are eight.

Plug in all the parameters in Supplementary Equation 11, we find that $\sigma_{internal}$ = 43.8 MPa. Combining the entropic stress of 20.5 MPa, the total internal stress due to programming is 64.3 MPa, which is close to the programming stress of 60 MPa. It has been known from polymer physics that both entropy and enthalpy, along with other factors, contribute to energy storage (*20*). Again, this very rough estimation confirms that, for this thermoset SMP, both entropy and enthalpy contribute to energy storage; however, with higher programming strain, enthalpy storage predominates.

Steric hindrance

To prove the argument of the steric effect, we take four steps. Step 1, based on the knowledge of organic chemistry and the chemical networks that have already been investigated, we assume that a certain group or groups provide the significant steric effect to the EPON-IPD network. Step 2, we find a diamine molecule with the exact or very similar structure but without the groups which are assumed to supply the steric hindrance. Step 3, we react the diamine with the EPON826 and obtain a thermoset network. Step 4, we test the thermal property, recovery stress and the energy storage mechanism to check if our argument of the steric effect is correct or not.

The first three steps are illustrated as the Supplementary Fig. 22. We assume that the groups providing the significant steric hindrance are the methyl groups in the IPD molecule including position one and position three (the ones with scissor). The ideal diamine is the molecule without these three methyl groups as shown in Supplementary Fig. 22. By searching the available and commercialized molecules, the 1,3-Bis (aminomethyl)cyclohexane (BACH) is chosen as the model diamine because it is a very similar molecule with the ideal structure but without the high steric hindrance (methyl groups); see Supplementary Fig. 22. To keep stoichiometry, the molar ratio of EPON and BACH is two to one.

In step 4, the thermal property of the synthesized EPON-BACH network is tested by DSC and the result is shown in Supplementary Fig. 23. The range of the glass transition is between 140°C and 150°C, which is a comparatively high glass transition range. This means that the EPON-

BACH network is also a rigid thermoset polymer. With the same method as that used for the EPON-IPD network, the thermoset polymer is compression programmed into 45% pre-strain as illustrated in Supplementary Fig. 24A. The recovery stress is also investigated and the result is shown in Supplementary Fig. 24B. The only difference here is the temperature for the programming and recovery which is 160°C, other than 170°C for the EPON-IPD network. The 160°C is 10 °C higher than the end-set point for the glass transition region for the EPON-BACH, which ensures that the programming and the recovery occur at the rubbery state for this thermoset polymer.

From Supplementary Fig. 24A, one can see that the maximum compressive stress (about 38 MPa) corresponding to the 45% pre-strain is lower than the EPON-IPD network, which is about 60 MPa, suggesting that the EPON-IPD network is stiffer. From Supplementary Fig. 24B, the recovery stress for the EPON-BACH is only about 8.5MPa which is much lower than the EPON-IPD network (17 MPa). This is a clear evidence that, the polymer network without the methyl groups cannot provide the steric hindrance and thus the recovery stress is much lower.

To verify the mechanism for the energy storage, the programmed EPON-BACH sample with the 45% pre-strain is characterized by DSC and the result is shown in Supplementary Fig. 25. Different from the EPON-IPD network, no inverse peak appears during the first heating cycle. It is proved that there is no enthalpy release during the free shape recovery process. Combining with the result of the recovery stress, it is concluded that the very similar thermoset network EPON-BACH, without the methyl groups attached on the cyclohexane structure in the diamine, cannot store energy in the form of enthalpy during the programming and the recovery stress is much lower than the EPON-IPD network, which consists of the methyl groups to provide the steric hindrance. Therefore, the argument on steric hindrance due to the methyl groups is valid.

Detailed explanation of the multiple energy well model

The concept of energy well against change of conformation is not a creation out of nothing. The potential energy changes by the rotational dihedral angle for butane and conformation for cyclohexane have been estimated for decades as the illustration shown in Supplementary Fig. 26. The butane can be treated as the smallest polyethylene which is a dimer. During the rotation of σ bond in the middle, the potential energy of the molecule fluctuates in a well-shape. When the methyl groups, which are electron rich groups in a butane, are closest to each other, the electron-repelling leads to the highest potential energy. The spatial position between chemical bonds is the

electron acceptable space (we can call it electron acceptor or electron hole). When the electron rich group is stabilized in the space lacking electrons, the total potential energy of the molecule is reduced. Once electron rich groups find the most comfortable positions as shown in Supplementary Fig. 26A- a and j, the potential energy touches the ground state. On the other hand, the stable positions that can still be found are local lowest potential energy states which are called metastable states as shown in Supplementary Fig. 26A- c and e. It is obvious that the potential energy of the metastable state is higher than the ground state, and more polymer repeat units form more metastable states. For example, the metastable state in butane is 3.8 kJ/mol higher than that of the ground state. The energy evolution by free rotation of chemical bonds was studied by Flory *(21)* and Tylor in 1940s (*22*). The multiple energy well model is based on this established knowledge. Nevertheless, some differences need to be pointed out. Firstly, the metastable position of bonds is not only affected by the intramolecular interaction like butane, but is also affected by the intermolecular interaction. In other words, the circumstance of the rotatable segments in a polymer network also affects the variation of the energy states. All interactions in molecular level can be generalized by electron repelling (peak of energy well) or electron stabilization (bottom of energy well) by electron acceptable space (electron acceptor) or electron vacancy space (electron hole). During the rotation of the chemical bonds, the local metastable position can be reached. The process of searching then staying at a metastable position can be imaged as the CSBs fall into an energy well. Secondly, both tension and compression cannot rotate the torsional angle to exceed the limit, which is 180 degrees. Therefore, during the programming of the polymer network, the pattern of potential energy is not symmetry as butane. Only half of the pattern can be revealed and it is kept ramping up.

Free shape recovery versus exothermic chemical reaction

The free shape recovery, as a spontaneous process associated with Gibbs free energy variation, has a lot of analogies compared with an exothermic chemical reaction as shown in Supplementary Fig. 27. The classical interpretation of a chemical reaction is described as follow. Although the free energy of reactants is higher than the product, the reaction will not occur without the activation energy. Before the spontaneous process happens, the reactants need to be excited into a high energetical level by heat, light, microwave or others. The total free energy will be stabilized by the variation of the molecular structure or degree of freedom. The Gibbs free energy of reactants is higher than the products and the free energy can be separated into enthalpy part and

entropy part. The enthalpic part is due to the type of chemical bonding that is changed. In shape memory effect, although the free energy of the fixed polymer network is higher than the original shape, it will not recover spontaneously without energy input. After the excitation by heating, the spontaneous transition will happen. The total energy is stabilized by the conformational and structural variation in the network during the recovering. The total free energy of the polymer network can also be separated into the enthalpic part and entropic part. The difference between these two phenomena is that the chemical bonds, regardless of reactants or products, exist naturally. The conformation or structure of the polymer network located at high energy state needs programming.

Recovery rate

The recovery rate of SMPs during free shape recovery is a significant property for all shape memory polymers. In this multiple energy well model, it corresponds to the time for the CSBs to roll down to the ground state. The free recovery process can be divided into two regions. The driving force for the high-energy region is the combination of entropy and enthalpy. In this region, the CSBs will be pulled back to low energy well by the stretched bonds. Subsequently, the CSBs located at the peak of an energy well is not in an equilibrium state. In this case, the driving force from the stretched bonds is the dominant factor for controlling shape recovering rate. In the low-energy region, the driving force that helps the CSBs fall back into low energy well is entropy only. If the chance of falling into an old or new energy well is equal, the frequency of CSBs vibrating in one energy well will determine the recovery rate. These characteristics is affected by the intrinsic property of the network, the environment of rotatable bond, and the temperature.

Recovery ratio

Although the multiple energy well model assumes that the polymer network contains no defect and no permanent deformation happens during the programming process, this model is capable of explaining the shape memory effect (SME) with plastic deformation by slight modification as shown in Supplementary Fig. 28A. Even for a perfect polymer network, permanent deformation can happen such as breaking chemical bonds due to over-programming. It happens much more easily for physically crosslinked SMPs because the network is constructed by chain entanglements or intermolecular interaction. The shape is hardly recovered when permanent deformation occurs. In this case, the term named shape recovery ratio is employed to define the recovered shape or strain quantitatively.

As shown in Supplementary Fig. 28B, energetic wells will break into discontinuous pieces if the permanent deformation happens. The energy absorbed when the SME is triggered will be consumed by the completed recovering part. If the rest of the energy is not able to overcome the energy gap formed by permanent deformation, the shape recovering will not happen for the residual shape (strain).

Supplementary References

1. G. Li. *Self-Healing Composites: Shape Memory Polymer Based Structures*. John Wiley & Sons, Inc., West Sussex, UK, (2014).
2. N. Lakhera, C. M. Yakachi, T. D. Nguyen, C. P. Frick. Partially constrained recovery of (meth)acrylate shape-memory polymer networks. *J. Appl. Polym. Sci*. **126**, 82-82 (2012).
3. Wang, G. Li, Stress memory of a thermoset shape memory polymer. *J. Appl. Polym. Sci*. **132**, 42112 (2015).
4. M. A. Di Prima, M. Lesniewski, K. Gall, D. L. McDowell, T. Sanderson, D. Campbell. Thermo-mechanical behavior of epoxy shape memory polymer foams. *Smart Mater. Struct.* **16**, 2330–2340 (2007).
5. ASM International. *Atlas of Stress-Strain Curves*-2nd ed. ASM International, OH, (2002).
6. E.L. Kirkby, J.D. Rule, V.J. Michaud, N.R. Sottos, S.R. White, J.A.E. Månson. Embedded shape-memory alloy wires for improved performance of self-healing polymers. *Adv. Funct. Mater*. **18**, 2253-2260, (2008).
7. C.L. Lewis, Y. Meng, and M. Anthamatten. Well-Defined Shape-Memory Networks with High Elastic Energy Capacity. *Macromolecules* **48**, 4918−4926, (2015).
8. P. M. Ajayan, L. S. Schadler, C. Giannaris, A. Rubio. Single-walled carbon nanotube–polymer composites: strength and weakness. *Adv. Mater*. **12**, 750-753 (2000).
9. D. Yang et al. Chemical analysis of graphene oxide films after heat and chemical treatments by X-Ray photoelectron and micro-Raman spectroscopy. *Carbon* **47**, 145-152 (2009).
10. G. Hähner. Near edge X-ray absorption fine structure spectroscopy as a tool to probe electronic and structural properties of thin organic films and liquids. *Chem. Soc. Rev*. **35**, 1244-1255 (2006).
11. Koprinarov, A. Lippitz, J. F. Friedrich, W. E. S. Unger, Ch. Wöll. Oxygen plasma induced degradation of the surface of poly(styrene), poly(bisphenol-A-carbonate) and poly(ethylene

terephthalate) as observed by soft X-ray absorption spectroscopy (NEXAFS). *Polymer* **39**, 3001-3009 (1998).

12. M. Mooney. A theory of large elastic deformation. *J. Appl. Phys.* **11**, 582-592 (1940).
13. M. Mooney. The thermodynamics of a strained elastomer. I. general analysis. *J. Appl. Phys.* **19**, 434-444 (1948).
14. M. Anthamatten, S. Roddecha, and J. Li. Energy Storage Capacity of Shape-Memory Polymers. *Macromolecules* **46**, 4230−4234 (2013).
15. C. C. Hornat, Y. Yang, M. W. Urban. Quantitative predictions of shape-memory effects in polymers. *Adv. Mater.* **29**, 1603334 (2017).
16. P. M. Morse. Diatomic molecules according to the wave mechanics. II. vibrational levels. *Phys. Rev.* **34**, 57-64 (1929).
17. K. Tashiro, G. Wu, M. Kobayashi. Quasiharmonic treatment of infrared and Raman vibrational frequency shifts induced by tensile deformation of polymer chains. *J. Polym. Sci. Part B* **28**, 2527-2553 (1990).
18. R. S. Bretzlaff, R. P. Wool. Frequency shifting and asymmetry in infrared bands of stressed polymers. *Macromolecules* **16**, 1907-1917 (1983).
19. Q. Wei, A.K. Sharma, J. Sankar, J. Narayan. Mechanical properties of diamond-like carbon composite thin films prepared by pulsed laser deposition. *Composites: Part B* **30**, 675–684 (1999).
20. J.J. Aklonis and W.J. MacKnight. *Introduction to Polymer Viscoelasticity* (2nd Ed.). John Wiley & Sons, (1983).
21. P. J. Flory. *Principles of polymer chemistry,* Cornell University Press, Ithaca, NY, (1953).
22. W.J. Taylor. Average length and radius of normal paraffin hydrocarbon molecules. *J. Chem. Phys.* **16**, 257-267 (1948).
